# Remdesivir Strongly Binds to RNA-Dependent RNA Polymerase, Membrane Protein, and Main Protease of SARS-CoV-2: Indication From Molecular Modeling and Simulations

**DOI:** 10.3389/fphar.2021.710778

**Published:** 2021-07-07

**Authors:** Faez Iqbal Khan, Tongzhou Kang, Haider Ali, Dakun Lai

**Affiliations:** ^1^ School of Electronic Science and Engineering, University of Electronic Science and Technology of China, Chengdu, China; ^2^ Faculty of Medicine, International Ala-Too University, Bishkek, Kyrgyzstan

**Keywords:** SARS-CoV-2, remdesivir, main protease, membrane proteins, RNA-dependent RNA polymerase

## Abstract

Development of new drugs is a time-taking and expensive process. Comprehensive efforts are being made globally toward the search of therapeutics against SARS-CoV-2. Several drugs such as remdesivir, favipiravir, ritonavir, and lopinavir have been included in the treatment regimen and shown effective results in several cases. Among the existing broad-spectrum antiviral drugs, remdesivir is found to be more effective against SARS-CoV-2. Remdesivir has broad-spectrum antiviral action against many single-stranded RNA viruses including pathogenic SARS-CoV and Middle East respiratory syndrome coronavirus (MERS-CoV). In this study, we proposed that remdesivir strongly binds to membrane protein (Mprotein), RNA-dependent RNA polymerase (RDRP), and main protease (Mprotease) of SARS-CoV-2. It might show antiviral activity by inhibiting more than one target. It has been found that remdesivir binds to Mprotease, Mprotein, and RDRP with −7.8, −7.4, and −7.1 kcal/mol, respectively. The structure dynamics study suggested that binding of remdesivir leads to unfolding of RDRP. It has been found that strong binding of remdesivir to Mprotein leads to decrease in structural deviations and gyrations. Additionally, the average solvent-accessible surface area of Mprotein decreases from 127.17 to 112.12 nm^2^, respectively. Furthermore, the eigenvalues and the trace of the covariance matrix were found to be low in case of Mprotease–remdesivir, Mprotein–remdesivir, and RDRP–remdesivir. Binding of remdesivir to Mprotease, Mprotein, and RDRP reduces the average motions in protein due to its strong binding. The MMPBSA calculations also suggested that remdesivir has strong binding affinity with Mprotein, Mprotease, and RDRP. The detailed analysis suggested that remdesivir has more than one target of SARS-CoV-2.

## Introduction

The flare-up of a surprising sickness showing extreme pneumonia and respiratory distress arose in Wuhan, China, in December 2019. Coronavirus disease 2019 (COVID-19) is brought about by severe acute respiratory syndrome coronavirus 2 (SARS-CoV-2), earlier named a novel coronavirus (2019-nCOV), and is a positive single-stranded RNA virus that has a place in the family Coronaviridae. The disease has spread across the boundary, and on March 11, 2020, the World Health Organization pronounced COVID-19 as a pandemic (Ahmed et al., 2020). The virus initially attacks the respiratory system and would cause flu-like symptoms such as cough and fever and, in severe conditions, leads to difficulty in breathing (Zou et al., 2020). According to statistical data, mortality is high in elderly (above 60 years of age) and in individuals with comorbid conditions. Apart from severe acute respiratory distress syndrome and respiratory failure, coronavirus disease (COVID-19) also manifests as systemic inflammation, acute cardiac injury, leading to sepsis, heart failure, and multiple organ dysfunction in patients at high risk (Wang D. et al., 2020).

Coronaviruses (CoVs) are a group of enveloped viruses, containing a non-segmented, positive-sense RNA genome, and pathogenic in nature (Xu Z. et al., 2020). SARS-CoV-2, the causative agent of COVID-19, has a more pathogenic form with respect to the previously mentioned SARS-CoV (2002) and Middle East respiratory syndrome coronavirus (MERS-CoV, 2013). To develop novel helpful therapeutic agents against SARS-CoV-2, it is important to understand the pathogenesis and virulence of virus (Chen et al., 2020).

Coronaviruses are the largest group of viruses belonging to the Coronaviridae family of order Nidovirales. Coronaviridae consists of two subfamilies, Coronavirinae and Torovirinae, respectively. Coronavirinae is further classified into four groups, the alpha, beta, gamma, and delta coronaviruses (Fehr and Perlman, 2015). Out of four groups, alpha and beta coronaviruses infect mammals, gamma-CoVs infect avian species, whereas delta coronaviruses infect both mammals and aves. MERS-CoV, SARS-CoV, and SARS-CoV-2 belong to beta coronaviruses that are transmitted through zoonotic transmission and spread among humans *via* close contact. The person-to-person spread of SARS-CoV-2, calculated by the primary reproduction number (R0), is about 2.6, which clearly indicates that the infected cases grow at an exponential rate (Chen et al., 2020; Wu et al., 2020).

CoVs particles are small (65–125 nm in diameter) and have the largest single-stranded RNA that is ∼26–32 kilobase (kb) long (Shereen et al., 2020). The genomic sequence of SARS-CoV-2 has 80% similarity to the genome of SARS-CoV and MERS-CoV, and more than 90% similarities have been found for structural proteins and essential enzymes. These important sequence similarities indicate a common pathogenesis mechanism and thus similar therapeutic targeting (Lau and Peiris, 2005). Coronaviruses contain four main structural proteins. These are the spike (S), membrane (M), envelope (E), and nucleocapsid (N) proteins. All structural proteins are encoded within the 3 end of the viral genome and share high sequence similarities to the sequence of the corresponding protein of MERS-CoV and SARS-CoV. SARS-CoV-2 spike (S) protein is essential for binding to the host cell-surface receptor for entry into the host cell (Delmas and Laude, 1990; Lau and Peiris, 2005; Beniac et al., 2006).

The spike protein has two subunits S1 and S2. The S1 subunit contains the receptor-binding domain (RBD) that binds to the angiotensin-converting enzyme 2 (ACE2) receptor located on the surface of the host cell, followed by the fusion of the S2 subunit to the cell membrane. SARS-CoV and SARS-CoV-2 use the ACE2 receptor, whereas MERS-CoV uses the dipeptidyl peptidase 4 receptor to bind with viral spike (S) protein (Zhang et al., 2020). SARS-CoV, MARS-CoV, and newly detected SARS-CoV-2 infection would cause severe respiratory symptoms and lead to death in comorbid conditions. The SARS-CoV genome shows high adaptive mutation and makes it more pathogenic and difficult for drug and vaccine inventions (Xu J. et al., 2020).

Among the existing broad-spectrum antiviral drugs, remdesivir is found to be more effective against SARS-CoV-2 and seems to have a more promising future. Remdesivir is a prodrug, unlike other nucleoside analogs, with broad-spectrum antiviral activity against many single-stranded RNA viruses including pathogenic SARS-CoV and Middle East respiratory syndrome coronavirus (MERS-CoV) (Wang M. et al., 2020).

Remdesivir is a metabolically active form (GS-441524) that works by inhibiting viral RNA-dependent RNA polymerase (RDRP) while evading proofreading by viral exoribonuclease, which leads to premature termination of viral RNA transcription and hence inhibits the spread of the virus as well as the production of viral RNA (Wang M. et al., 2020). In this study, we proposed that remdesivir possibly binds to more than one target of SARS-CoV-2. We showed possible binding and inhibitory mechanisms of remdesivir on the C-terminal domain of SARS-CoV-2 nucleocapsid protein (CTD), envelope protein (Eprotein), main protease (Mprotease), membrane protein (Mprotein), N-terminal domain of nucleocapsid phosphoprotein (NTD), RDRP, and spike protein (Sprotein) using several computational approaches. It shows strong inhibitory effects on Mprotein, RDRP, and Mprotease of SARS-CoV-2.

## Materials and Methods

### 3D Structure Modeling

The crystal structures of CTD nucleocapsid (PDB: 7c22), Mprotease (PDB: 7k40), NTD nucleocapsid (PDB: 7acs), RDRP (PDB: 6m71), and Sprotein (PDB: 6vsb) were obtained from the PDB (https://www.rcsb.org/), and the structure of remdesivir was obtained from PubChem (CID 121304016). Because the crystal structures of Eprotein and Mprotein are not solved yet and published in the PDB, I-Tasser which is a graded protocol for the prediction of structure and function of the protein was used to predict the structure of these two proteins (Roy et al., 2010). The missing residues from the crystal structure were modeled using MODELLER software (Webb and Sali, 2016). The comprehensive procedures are mentioned in previous communication (Khan et al., 2015; Khan et al., 2016; Khan et al., 2017). Discovery Studio and PyMOL were used for visualizing and drawing structures.

### Docking Studies

The molecular docking of remdesivir with CTD, Eprotein, Mprotease, Mprotein, NTD, RDRP, and Sprotein was performed using AutoDock Vina (Trott and Olson, 2010). Each of the proteins was individually prepared for docking using standard protocols (Khan et al., 2020a; Khan et al., 2020b; Khan et al., 2021). The finest poses of each complex were selected on the foundation of binding energy as well as proper orientation of remdesivir into the active pockets of target proteins (Schmidt et al., 2018).

### MD Simulations

Several 100 ns MD simulations were executed on CTD, CTD–remdesivir, Eprotein, Eprotein–remdesivir, Mprotease, Mprotease–remdesivir, Mprotein, Mprotein–remdesivir, NTD, NTD–remdesivir, RDRP, RDRP–remdesivir, Sprotein, and Sprotein–remdesivir by GROMACS 2018.2 (Van Der Spoel et al., 2005; Naz et al., 2018; Ali et al., 2019; Beg et al., 2019; Gulzar et al., 2019b; Mohammad et al., 2019). The PRODRG server (Schuttelkopf and van Aalten, 2004) was used to obtain the parameters and topology files for the remdesivir molecule using a standard protocol (Camilloni et al., 2008; Carr et al., 2013; Syed et al., 2018a). Similarly, a reasonable quantity of Na^+^ and Cl^−^ ions was added to preserve neutrality of the system. The final production stage of 100 ns was achieved at 298 K. The comprehensive MD simulation methodology is mentioned in previous communications (Syed et al., 2018a; Gulzar et al., 2019a; Furkan et al., 2019; Qausain et al., 2020).

### Essential Dynamics

Principal component analysis (PCA) or essential dynamics (ED) was calculated for each simulated system of CTD, CTD–remdesivir, Eprotein, Eprotein–remdesivir, Mprotease, Mprotease–remdesivir, Mprotein, Mprotein–remdesivir, NTD, NTD–remdesivir, RDRP, RDRP–remdesivir, Sprotein, and Sprotein–remdesivir. It is calculated by the diagonalization of the covariance matrix C, with the elements, explained as follows:

$$C_{ij}=<\left(r_{i}\;-\;\langle r_{i}\rangle \right)\times \left(r_{j}\;-\;\langle r_{j}\rangle \right)\;\left(i,\;j\;=\;1,2,3,\&,3N\right).$$ (1)

Here, *r*_*i*_ represents the Cartesian coordinate of the *i*th Cα atom, *N* represents the number of Cα atoms, and <*r*_*i*_> signifies the time average over all the configurations (Khan et al., 2020b; Durrani et al., 2020; Hassan et al., 2020).

### Gibbs Free Energy Landscape

The GFE landscape offers conformational variations in proteins (Khan et al., 2016; Syed et al., 2018b; Gupta et al., 2020a; Gupta et al., 2020b). To get 2D and 3D depiction, the GFE landscapes were projected onto PC1 and PC2 for CTD, CTD–remdesivir, Eprotein, Eprotein–remdesivir, Mprotease, Mprotease–remdesivir, Mprotein, Mprotein–remdesivir, NTD, NTD–remdesivir, RDRP, RDRP–remdesivir, Sprotein, and Sprotein–remdesivir during 100 ns MD simulations. It is mentioned as follows:

$$G_{\left(\text{PC}1\text{, PC}2\right)}\;=-k_{\text{B}}T\ln P_{\left(\text{PC}1\text{, PC}2\right)}.$$ (2)

Here, *k*_B_ represents the Boltzmann constant, *T* represents the temperature, and *P*_(*PC1*, *PC2*)_ represents the normalized joint probability distribution.

## Results and Discussion

The SARS-CoV-2 genome is made of 29,891 nucleotides with 9,860 amino acids (Chan et al., 2020), packaged in a circular nucleocapsid protein and encapsulated by envelope proteins (Li, 2016). All proteins included in this study, namely, CTD, Eprotein, Mprotease, Mprotein, NTD, RDRP, and Sprotein, play significant roles and hence are promising therapeutic targets to control COVID-19 (Satarker and Nampoothiri, 2020). Previously, it has been mentioned in the literature that remdesivir inhibits viral RDRP protein, but some literature suggested that it inhibits more than one targeted protein of SARS-CoV-2 (Nguyen et al., 2020). In the present *in silico* study, we focussed on the inhibitory mechanism of remdesivir on several SARS-CoV-2 targets.

### Interaction of Remdesivir With SARS-CoV-2 Proteins

**FIGURE 1:**
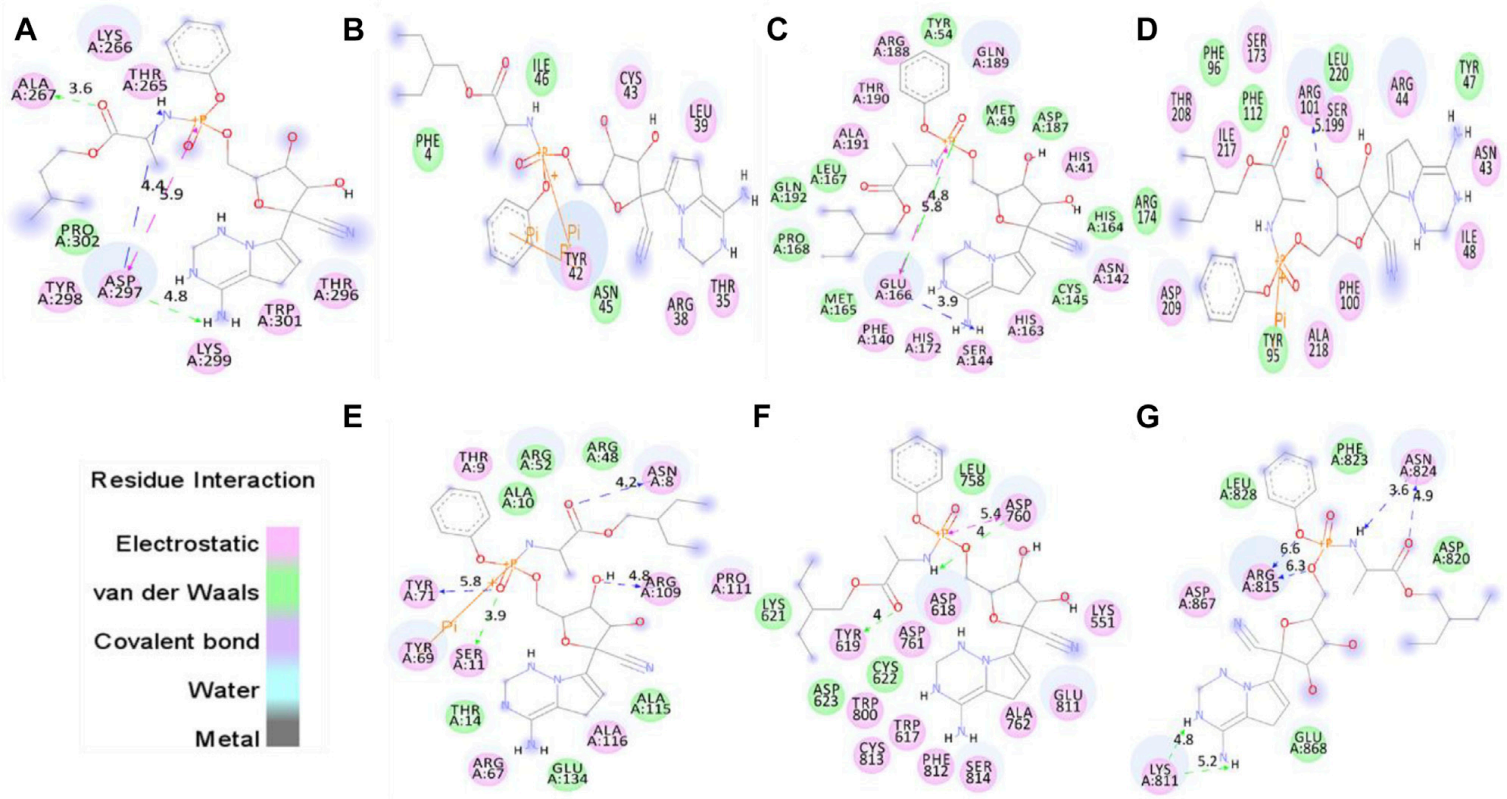
Molecular docking of remdesivir into the active pocket of CTD, Eprotein, Mprotease, Mprotein, NTD, RDRP, and Sprotein, respectively. The structure indicated different residual interactions in **(A)** CTD–remdesivir, **(B)** Eprotein–remdesivir, **(C)** Mprotease–remdesivir, **(D)** Mprotein–remdesivir, **(E)** NTD–remdesivir, **(F)** RDRP–remdesivir, and **(G)** Sprotein–remdesivir, respectively. The electrostatic, van der Waals, and covalent bonds are represented in pink, green, and purple colors, respectively.

The proper orientation of remdesivir in the active pockets of CTD, Eprotein, Mprotease, Mprotein, NTD, RDRP, and Sprotein has been monitored, and active site residues were targeted. Remdesivir binds with CTD, Eprotein, Mprotease, Mprotein, NTD, RDRP, and Sprotein with −4.8, −4.2, −7.8, −7.4, −6.2, −7.1, and −5.8 kcal/mol, respectively (Figure 1). It shows several electrostatic and van der Waals interactions as listed in Table 1. Remdesivir shows weak van der Waals interactions with CTD and Eprotein. It shows strong binding affinity with Mprotease, Mprotein, and RDRP. The important residues that interacted with inhibitor N3 in Mprotease are Thr24, Thr25, Thr26, His41, Met49, Phe140, Leu141, Asn142, Gly143, Ser144, Cys145, His163, His164, Met165, Glu166, Pro168, His172, Asp187, Gln189, Thr190, Ala191, and Gln192 (Jin et al., 2020). We found that remdesivir binds in the same pocket and shows interactions with residues His41, Met49, Tyr54, Phe140, Asn142, Ser144, Cys145, His163, His164, Met165, Glu166, Leu167, Pro168, His172, Asp187, Arg188, Gln189, Thr190, Ala191, and Gln192.

**TABLE 1:** Molecular docking of remdesivir with CTD, Eprotein, Mprotease, Mprotein, NTD, RDRP, and Sprotein, respectively.

S. No.	Proteins	Electrostatic interactions	van der Waals interactions	Energy (kcal/mol)
1	CTD–remdesivir	Thr265, Lys266, Ala267, Thr296, Asp297, Tyr298, Lys299, and Trp301	Pro302	-4.8
2	Eprotein–remdesivir	Thr35, Arg38, Leu39, Tyr42, and Cys43	Phe4, Asn4, and Ile46	-4.2
3	Mprotease–remdesivir	His41, Phe140, Asn142, Ser144, His163, Glu166, His172, Arg188, Gln189, Thr190, and Ala191	Met49, Tyr54, Cys145, His164, Met165, Leu167, Pro168, Asp187, and Gln192	-7.8
4	Mprotein–remdesivir	Asn43, Arg44, Ile48, Phe100, Arg101, Ser173, Ser199, Thr208, Asp209, Ile217, and Ala218	Tyr47, Tyr95, Phe96, Phe112, Arg174, and Leu220	-7.4
5	NTD–remdesivir	Asn8, Thr9, Ser11, Arg67, Tyr69, Tyr71, Arg109, Pro111, and Ala116	Ala10, Thr14, Arg48, Arg52, Ala115, and Glu134	-6.2
6	RDRP–remdesivir	Lys551, Trp617, Asp618, Tyr619, Asp760, Asp761, Ala762, Trp800, Glu811, Phe812, Lys813, and Ser814	Lys621, Lys622, Asp623, and Leu758	-7.1
7	Sprotein–remdesivir	Lys811, Arg815, Asn824, and Asp867	Asp820, Phe823, Leu828, and Glu868	-5.8

It has been reported that the targeting of active sites of RDRP such as Asp760 and Asp761 by antiviral drugs could be a potential therapeutic option for inhibition of viral replication (Aftab et al., 2020). Galidesivir, a potential inhibitor of SARS-CoV-2, which binds strongly to the active site of RDRP shows interactions with residues Asp760, Asp761, Gly616, Trp617, Asp618, Tyr619, Pro620, Lys621, Cys622, Leu758, Ser759, Ala762, Ala797, Lys798, Cys799, Trp800, His810, Glu811, Phe812, Cys813, Ser814, and Gln815 (Aftab et al., 2020). We found that remdesivir binds strongly in the same active pocket and interacts with residues Lys551, Trp617, Asp618, Tyr619, Lys621, Lys622, Asp623, Leu758, Asp760, Asp761, Ala762, Trp800, Glu811, Phe812, Lys813, and Ser814. In case of Mprotein, the predicted active site residues are Ile48, Trp92, Tyr95, Phe96, Ile97, Ser99, Phe100, and Ala104 (Zhang et al., 2017). Remdesivir binds to one of the pockets of Mprotein and interacts with residues Asn43, Arg44, Tyr47, Ile48, Tyr95, Phe96, Phe100, Arg101, Phe112, Ser173, Arg174, Ser199, Thr208, Asp209, Ile217, Ala218, and Leu220. The protein–drug complexes were further analyzed for several 100 ns MD simulations.

### Structural Dynamics of CTD

**FIGURE 2:**
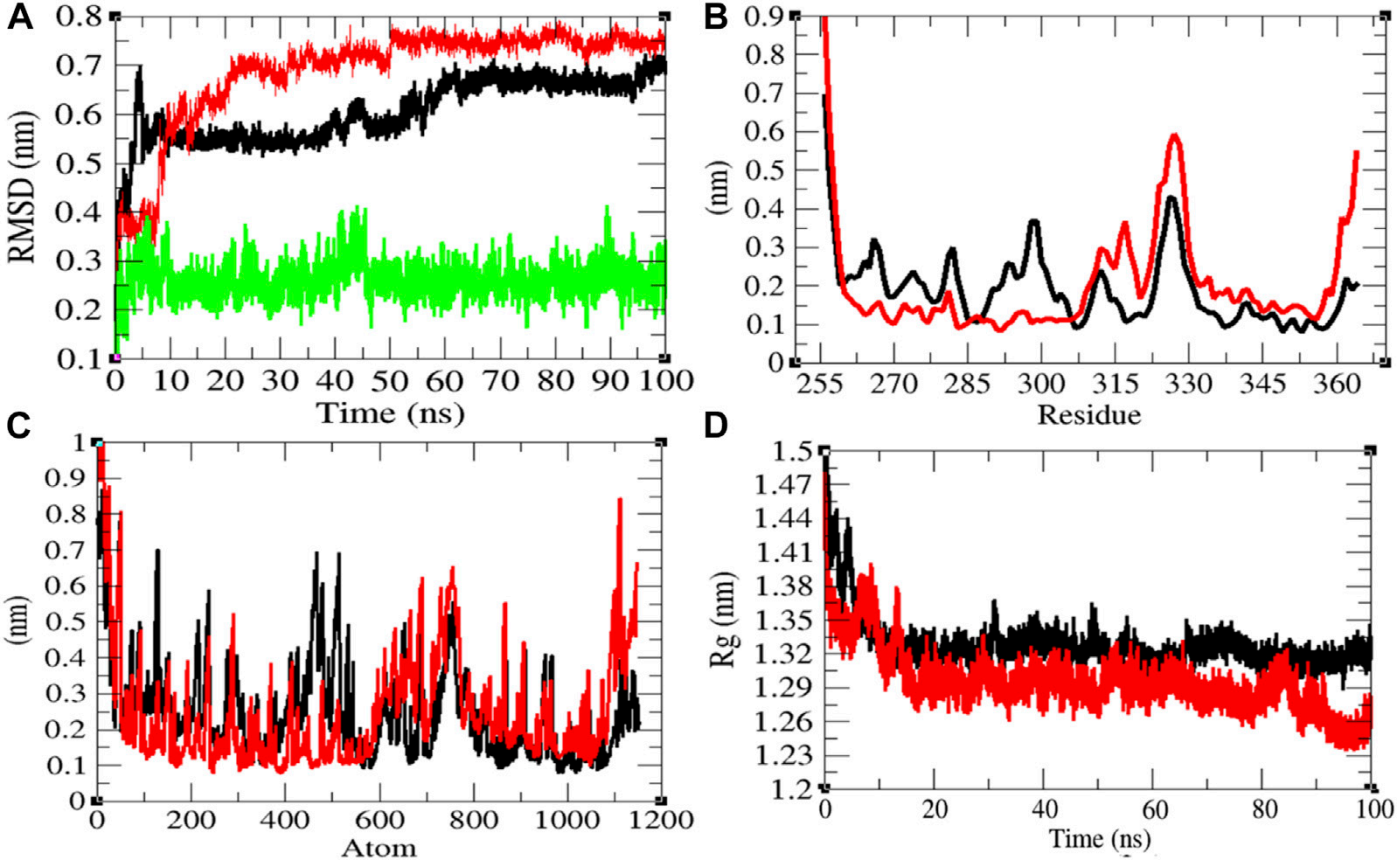
Structural dynamics of CTD. **(A)** Root mean square deviation plot for CTD (black), CTD–remdesivir (red), and remdesivir (green) as a function of time. **(B)** Root mean square fluctuations vs. residues. **(C)** Root mean square fluctuations vs. atoms. **(D)** Radius of the gyration (*R*_g_) plot.

In order to explore the structural dynamics of CTD, CTD–remdesivir, Eprotein, Eprotein–remdesivir, Mprotease, Mprotease–remdesivir, Mprotein, Mprotein–remdesivir, NTD, NTD–remdesivir, RDRP, RDRP–remdesivir, Sprotein, and Sprotein–remdesivir, the root mean square deviation (RMSD), the root mean square fluctuation (RMSF), and the radius of gyration (*R*_*g*_) were analyzed (Kuzmanic and Zagrovic, 2010). The average RMSD values of CTD and CTD–remdesivir were found to be 0.60 and 0.69 nm, respectively. Additionally, remdesivir was found to have constant fluctuations in the active pocket of CTD (Figure 2A). It has been found that RMSD values slightly increase upon binding of remdesivir. There was a rise in residual and atomic fluctuations of CTD also reported due to binding of remdesivir (Figures 2B,C). The average *R*_*g*_ values for CTD and CTD–remdesivir were found to be 1.33 and 1.29 nm, respectively (Figure 2D). It was found that CTD–remdesivir has more tight packing than CTD alone due to binding of remdesivir. The results from above analysis clearly state that binding of remdesivir in the active pocket of CTD slightly changes the structure dynamics of protein.

The solvent-accessible surface area (SASA) is explained as the surface area of a protein which forms networks with its solvent molecules (Mazola et al., 2015). The average SASA values with respect to protein for CTD and CTD–remdesivir were found to be 61.46 and 56.69 nm^2^, respectively. The SASA plot suggested that internal residues in CTD are not exposed to solvent when remdesivir binds to it. Furthermore, the free energy of solvation for CTD and CTD–remdesivir was found to be 145.89 and 126.21 kJ/mol/nm^2^, respectively.

The purpose of secondary structure is to spot the structural features of proteins. The secondary structure assignments in CTD and CTD–remdesivir such as the β-sheet, α-helix, and turn were split into separate residues in each period. The average number of residues that participated in secondary structure formation of CTD and CTD–remdesivir was compared (Table 2). It was found that the total average residues that contributed to structure development in case of CTD and CTD–remdesivir were found to be 42 and 49%, respectively. It has been found that CTD is not inhibited or unfolded upon binding of remdesivir. In other words, remdesivir did not show inhibitory effects on CTD. It was also found that the volume of CTD and CTD–remdesivir was 23.07 and 21.65 nm^3^, respectively. The average density of CTD and CTD–remdesivir was found to be 890.60 and 949.17 g/L, respectively (Supplementary Figure S1).

**TABLE 2:** Percentage of residues in CTD, CTD–remdesivir, Eprotein, Eprotein–remdesivir, Mprotease, Mprotease–remdesivir, Mprotein, Mprotein–remdesivir, NTD, NTD–remdesivir, RDRP, RDRP–remdesivir, Sprotein, and Sprotein–remdesivir, which participated in average structure formation during MD simulations.

Protein	Percentage of secondary structure (SS %)							
	Structure^a^	Coil	β-Sheet	β-Bridge	Bend	Turn	α-Helix	3_10_-Helix
CTD	42	38	8	2	20	10	22	1
CTD–remdesivir	49	30	2	3	19	18	26	1
Eprotein	36	41	1	3	24	5	26	0
Eprotein–remdesivir	42	35	2	5	23	10	25	0
Mprotease	60	27	25	2	11	9	25	2
Mprotease–remdesivir	62	27	26	2	10	10	24	1
Mprotein	46	34	10	2	19	12	22	1
Mprotein–remdesivir	46	30	10	3	24	12	21	0
NTD	42	41	25	4	16	13	0	1
NTD–remdesivir	41	40	26	4	18	9	1	1
RDRP	61	24	14	2	13	9	36	1
RDRP–remdesivir	59	26	12	2	13	9	35	2
Sprotein	59	25	28	1	14	8	22	1
Sprotein–remdesivir	61	23	31	2	15	8	20	0

a Structure = α-helix + β-sheet + β-bridge + turn.

### Structural Dynamics of Eprotein

**FIGURE 3:**
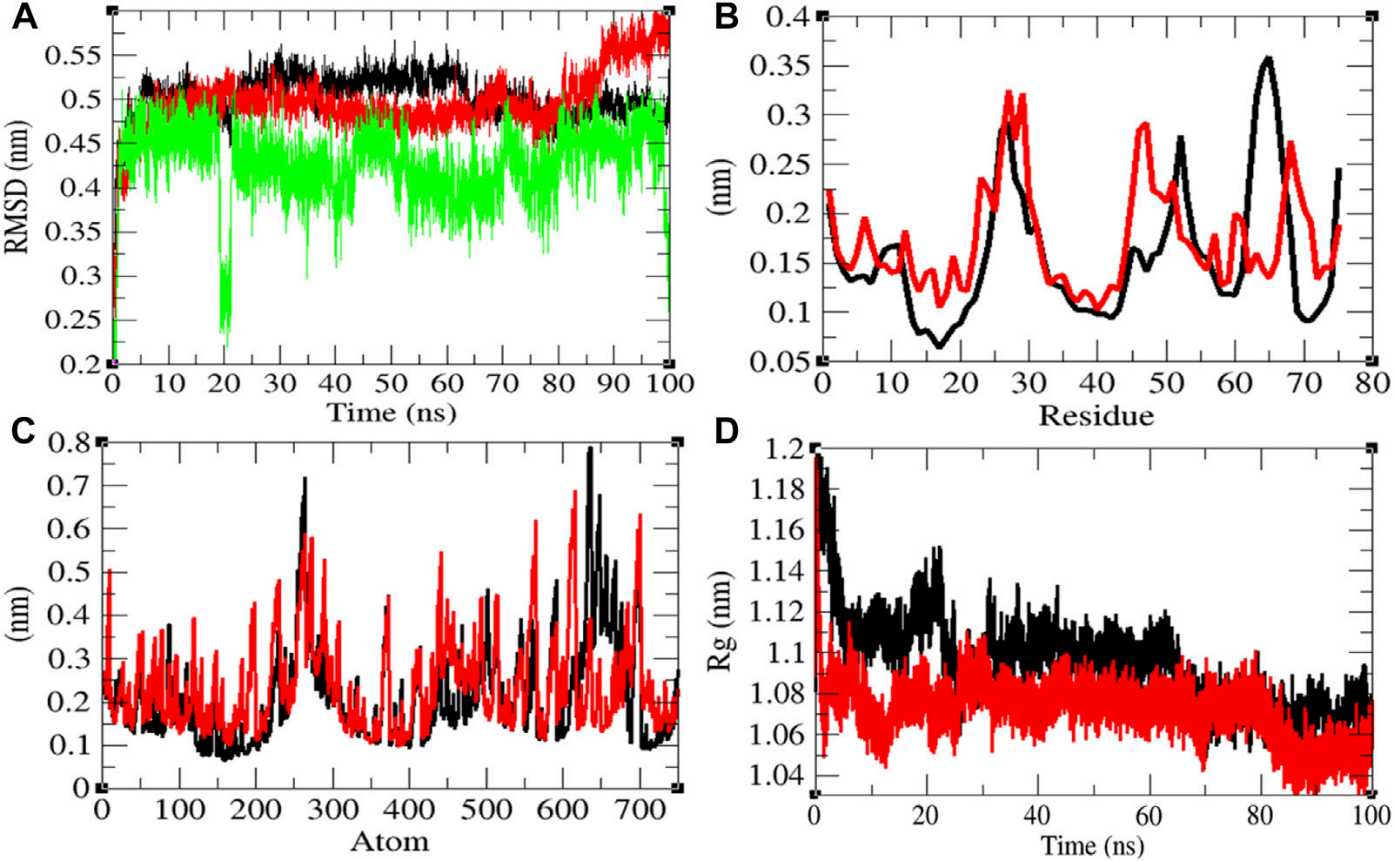
Structural dynamics of Eprotein. **(A)** Root mean square deviation plot for Eprotein (black), Eprotein–remdesivir (red), and remdesivir (green) as a function of time. **(B)** Root mean square fluctuations vs. residues. **(C)** Root mean square fluctuations vs. atoms. **(D)** Radius of the gyration (*R*_g_) plot.

The average RMSD values of Eprotein and Eprotein–remdesivir were found to be 0.50 and 0.49 nm, respectively. Remdesivir was found to have random fluctuations in the active pocket of Eprotein (Figure 3A). It has been found that average RMSD values were almost the same upon binding of remdesivir. There were some residual and atomic fluctuations reported due to binding of remdesivir (Figures 3B,C). The average *R*_*g*_ values for Eprotein and Eprotein–remdesivir were found to be 1.09 and 1.07 nm, respectively (Figure 3D). It was found that Eprotein–remdesivir has more tight packing than Eprotein alone due to binding of remdesivir.

The average SASA values with respect to protein for Eprotein and Eprotein–remdesivir were found to be 41.37 and 39.98 nm^2^, respectively. The SASA plot suggested that internal residues in Eprotein are not exposed to solvent when remdesivir binds to it. Furthermore, the free energy of solvation for Eprotein and Eprotein–remdesivir was found to be 134.93 and 119.30 kJ/mol/nm^2^, respectively. It was found that the total average residues that contributed to structure development in case of Eprotein and Eprotein–remdesivir were found to be 36 and 42%, respectively. It has been found that Eprotein is not inhibited or unfolded upon binding of remdesivir. In other words, remdesivir did not show inhibitory effects on Eprotein. It was also found that the volume of Eprotein and Eprotein–remdesivir was 15.11 and 15.13 nm^3^, respectively. The average density of Eprotein and Eprotein–remdesivir was found to be 919.62 and 918.27 g/L, respectively (Supplementary Figure S2). The volume and the density remain the same in both the cases.

### Structural Dynamics of Mprotease

**FIGURE 4:**
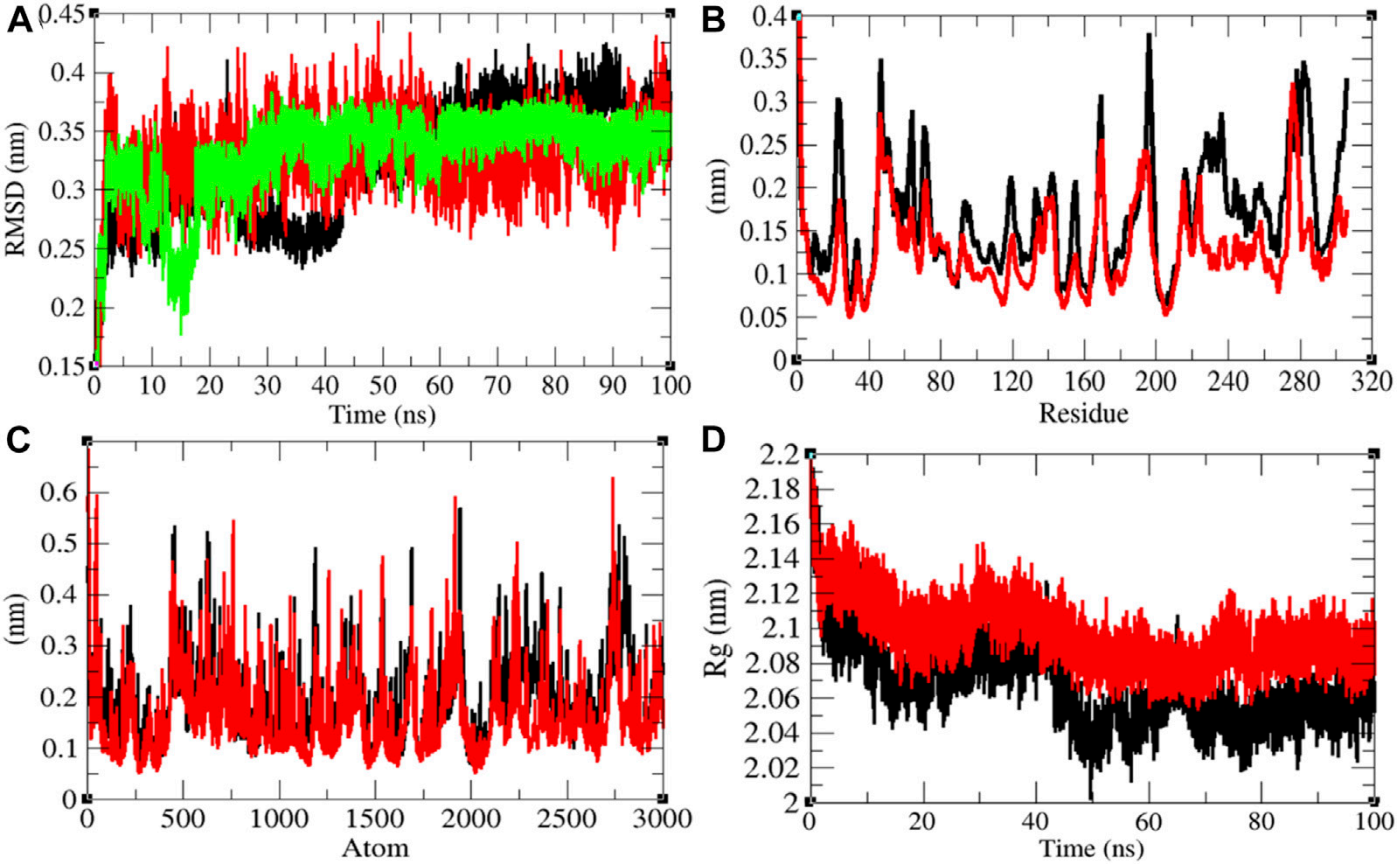
Structural dynamics of Mprotease. **(A)** Root mean square deviation plot for Mprotease (black), Mprotease–remdesivir (red), and remdesivir (green) as a function of time. **(B)** Root mean square fluctuations vs. residues. **(C)** Root mean square fluctuations vs. atoms. **(D)** Radius of the gyration (*R*_g_) plot.

Mprotease plays a key role in coordinating viral replication and transcription of the virus life cycle. It cleaves the major part of polyproteins and releases proteins that have replicative function. Therefore, Mprotease is a prime target of drugs for SARS-CoV-2 (Liu et al., 2020; Naik et al., 2020). The average RMSD values of Mprotease and Mprotease–remdesivir were found to be 0.32 and 0.33 nm, respectively. Remdesivir was found to have constant fluctuations in the active pocket of Mprotease (Figure 4A). It has been found that average RMSD values were the same upon binding of remdesivir. There were least residual and atomic fluctuations reported due to binding of remdesivir (Figures 4B,C). The average *R*_*g*_ values for Mprotease and Mprotease–remdesivir were found to be 2.07 and 2.10 nm, respectively (Figure 4D). It was found that Mprotease–remdesivir has more loose packing than Mprotease due to binding of remdesivir. This might be due to unfolding of Mprotease.

The average SASA values with respect to protein for Mprotease and Mprotease–remdesivir were found to be 155.43 and 156.34 nm^2^, respectively. The SASA plot suggested that internal residues in Mprotease are not much exposed to solvent when remdesivir binds to it. Furthermore, the free energy of solvation for Mprotease and Mprotease–remdesivir was found to be 207.35 and 213.14 kJ/mol/nm^2^, respectively. It was found that the total average residues that contributed to structure development in case of Mprotease and Mprotease–remdesivir were found to be 60 and 62%, respectively. It has been found that remdesivir has less impact on structural unfolding of Mprotease. It was also found that the volume of Mprotease and Mprotease–remdesivir was 54.98 and 55.10 nm^3^, respectively. The average density of Mprotease and Mprotease–remdesivir was found to be 1,020.79 g/L and 1,018.49 g/L, respectively (Supplementary Figure S3).

### Structural Dynamics of Mprotein

**FIGURE 5:**
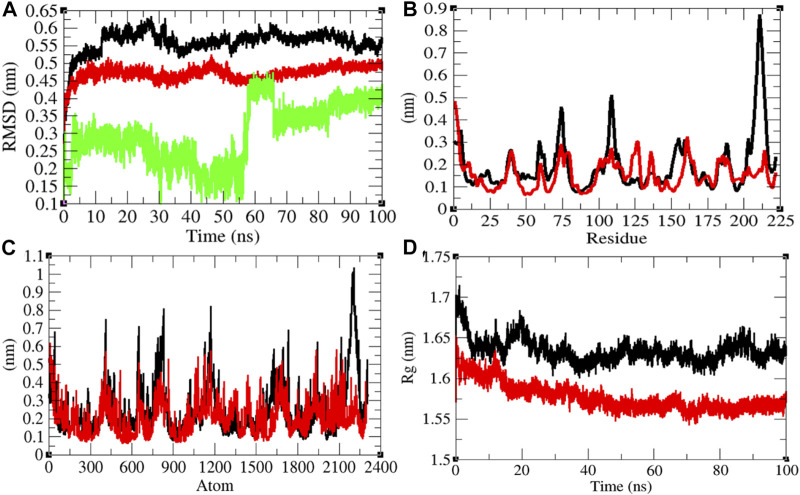
Structural dynamics of Mprotein. **(A)** Root mean square deviation plot for Mprotein (black), Mprotein–remdesivir (red), and remdesivir (green) as a function of time. **(B)** Root mean square fluctuations vs. residues. **(C)** Root mean square fluctuations vs. atoms. **(D)** Radius of the gyration (*R*_g_) plot.

The average RMSD values of Mprotein and Mprotein–remdesivir were found to be 0.56 and 0.47 nm, respectively. Remdesivir showed constant fluctuations in the active pocket of Mprotein (Figure 5A). It has been found that average RMSD values were less upon binding of remdesivir. This indicates that remdesivir binds tightly with Mprotein and possibly inhibits it. The residual and atomic fluctuations were also less due to binding of remdesivir (Figures 5B,C). The average *R*_*g*_ values for Mprotein and Mprotein–remdesivir were found to be 1.63 and 1.58 nm, respectively (Figure 5D). It was found that Mprotein–remdesivir has more tight packing than Mprotein alone. This might be due to strong binding of remdesivir with Mprotein.

The average SASA values with respect to protein for Mprotein and Mprotein–remdesivir were found to be 127.17 and 112.12 nm^2^, respectively. The SASA plot suggested that internal residues in Mprotein are not exposed to solvent when remdesivir binds to it. Furthermore, the free energy of solvation for Mprotein and Mprotein–remdesivir was found to be 237.24 and 205.11 kJ/mol/nm^2^, respectively. It was found that the total average residues that contributed to structure development in case of Mprotein and Mprotein–remdesivir were found to be the same (46%). It was also found that the volume of Mprotein and Mprotein–remdesivir was 45.70 and 42.34 nm^3^, respectively. The average density of Mprotein and Mprotein–remdesivir was found to be 914.05 and 986.80 g/L, respectively (Supplementary Figure S4).

The role of Mprotein of SARS-CoV-2 during host infection is not clearly understood. It was found that Mprotein binds with nucleocapsid protein and promotes completion of viral assembly by stabilizing the nucleocapsid protein and RNA complex (Astuti and Ysrafil, 2020). Thomas (2020) predicted that Mprotein of SARS-CoV-2 is structurally similar to SemiSWEET sugar transport proteins of prokaryotes (Thomas, 2020). He hypothesized that the SemiSWEET sugar transporter–like structure of Mprotein influences glycosylation of Sprotein. The SemiSWEET sugar transporter–like structure of Mprotein may be involved in multiple functions that may aid in the rapid proliferation, replication, and immune evasion of the SARS-CoV-2 virus.

### Structural Dynamics of NTD

**FIGURE 6:**
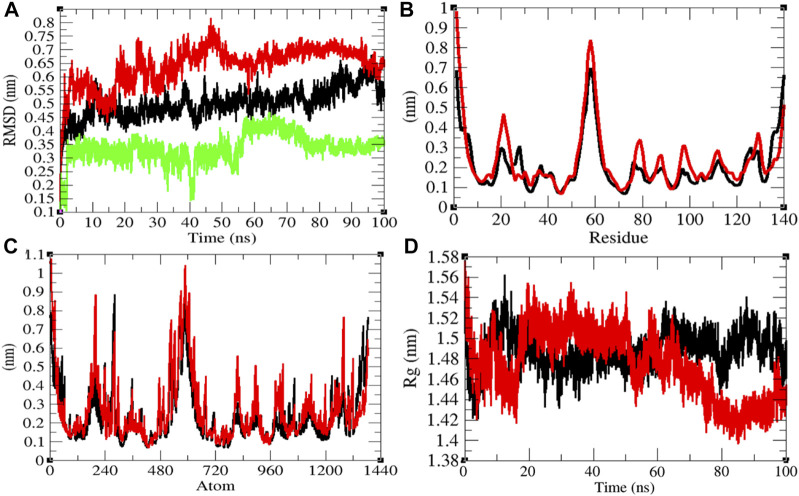
Structural dynamics of NTD. **(A)** Root mean square deviation plot for NTD (black), NTD–remdesivir (red), and remdesivir (green) as a function of time. **(B)** Root mean square fluctuations vs. residues. **(C)** Root mean square fluctuations vs. atoms. **(D)** Radius of the gyration (*R*_g_) plot.

The average RMSD values of NTD and NTD–remdesivir were found to be 0.50 and 0.64 nm, respectively. Additionally, remdesivir showed random fluctuations in the active pocket of NTD (Figure 6A). It has been found that average RMSD values were more upon binding of remdesivir. This indicates that the random fluctuations increased upon remdesivir binding to NTD. There was minor increase in residual and atomic fluctuations too (Figures 6B,C). The average *R*_*g*_ values for NTD and NTD–remdesivir were found to be 1.49 and 1.47 nm, respectively (Figure 6D). It was found that NTD–remdesivir has slightly more tight packing than NTD alone.

The average SASA values with respect to protein for NTD and NTD–remdesivir were found to be 74.51 and 75.73 nm^2^, respectively. The SASA plot suggested that internal residues in NTD are slightly exposed to solvent when remdesivir binds to it. Furthermore, the free energy of solvation for NTD and NTD–remdesivir was found to be 147.53 and 158.73 kJ/mol/nm^2^, respectively. The free energy of solvation for NTD–remdesivir was found to be higher than that of NTD. It was found that the total average residues that contributed to structure development in case of NTD and NTD–remdesivir were found to be 42 and 41%, respectively. It has been found that binding of remdesivir has minor effects on NTD. It was also found that the volume of NTD and NTD–remdesivir was 26.43 and 26.51 nm^3^, respectively. The average density of NTD and NTD–remdesivir was found to be 950.06 and 947.03 g/L, respectively. The volume and density of NTD remain almost the same in both the cases. There was no major impact of remdesivir on the structural volume of NTD during the course of simulations (Supplementary Figure S5).

### Structural Dynamics of RNA-Dependent RNA Polymerase

**FIGURE 7:**
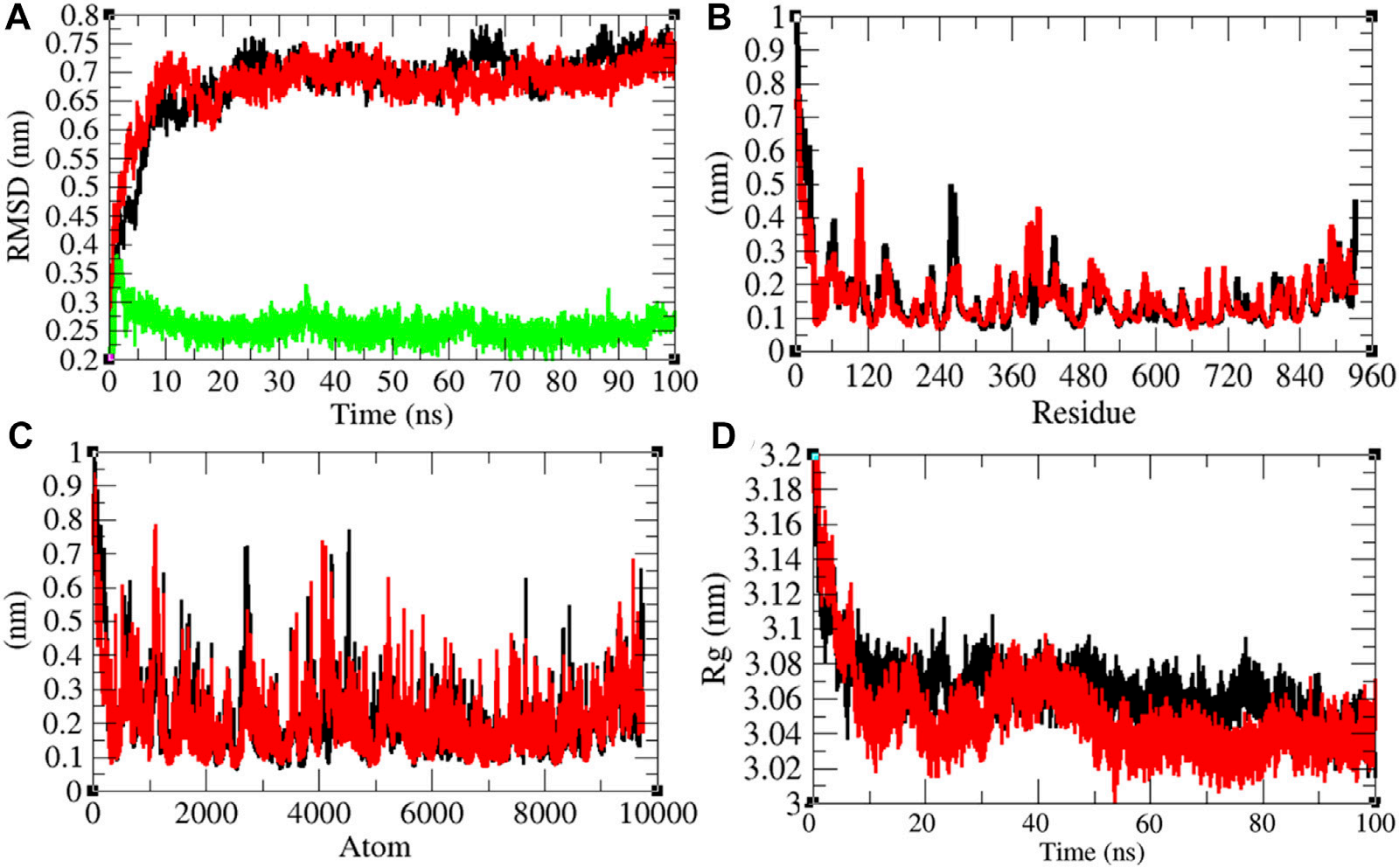
Structural dynamics of RDRP. **(A)** Root mean square deviation plot for RDRP (black), RDRP–remdesivir (red), and remdesivir (green) as a function of time. **(B)** Root mean square fluctuations vs. residues. **(C)** Root mean square fluctuations vs. atoms. **(D)** Radius of the gyration (*R*_g_) plot.

It has been reported that remdesivir inhibits RDRP and shows antiviral activity against multiple variants of the Ebola virus (Warren et al., 2016). *In vitro* experiments showed that remdesivir is effective against SARS-CoV and MERS-CoV by interfering with the polymerase function of RDRP (Agostini et al., 2018). It is also expected that remdesivir inhibits RDRP of SARS-CoV-2. The average RMSD values of RDRP and RDRP–remdesivir were found to be 0.68 and 0.67 nm, respectively. Additionally, remdesivir was stable in the active pocket of RDRP (Figure 7A). It has been found that the structure of RDRP was not deviated upon binding of remdesivir. The residual and atomic fluctuations were also less (Figures 7B,C). The average *R*_*g*_ values for RDRP and RDRP–remdesivir were found to be 3.06 and 3.05 nm, respectively (Figure 7D). It was found that RDRP–remdesivir has similar structural packing to RDRP alone.

The average SASA values with respect to protein for RDRP and RDRP–remdesivir were found to be 461.72 and 466.09 nm^2^, respectively. The SASA plot suggested that internal residues in RDRP are exposed to solvent when remdesivir binds to it. Furthermore, the free energy of solvation for RDRP and RDRP–remdesivir was found to be 479.17 and 517.78 kJ/mol/nm^2^, respectively. It was found that the total average residues that contributed to structure development in case of RDRP and RDRP–remdesivir were found to be 61 and 59%, respectively. It has been found that RDRP is slightly unfolded upon binding of remdesivir. It was also found that the volume of RDRP and RDRP–remdesivir was 169.68 and 169.68 nm^3^, respectively. The average density of RDRP and RDRP–remdesivir was found to be 1,043.81 and 1,043.83 g/L, respectively (Supplementary Figure S6).

### Structural Dynamics of Sprotein

**FIGURE 8:**
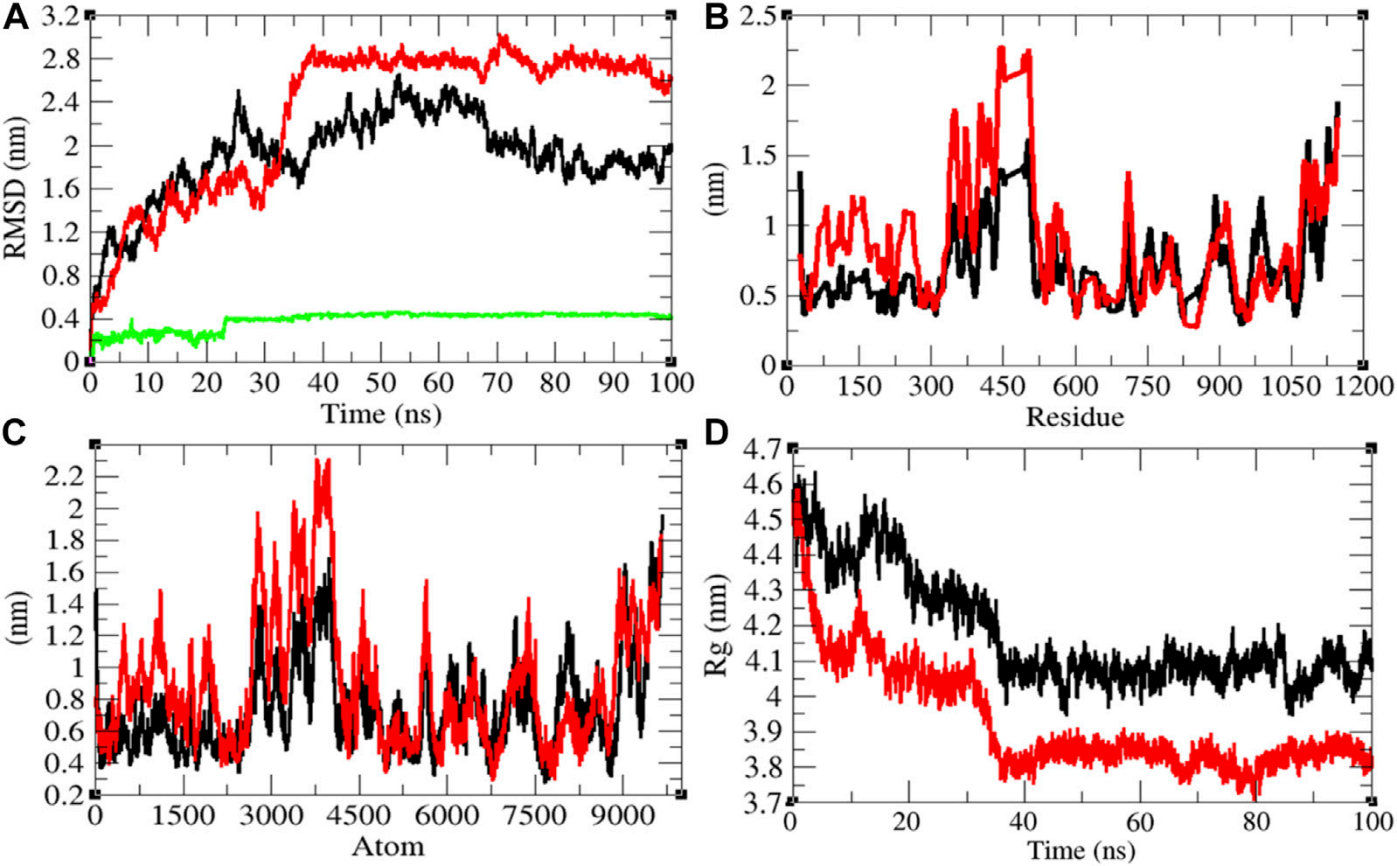
Structural dynamics of Sprotein. **(A)** Root mean square deviation plot for Sprotein (black), Sprotein–remdesivir (red), and remdesivir (green) as a function of time. **(B)** Root mean square fluctuations vs. residues. **(C)** Root mean square fluctuations vs. atoms. **(D)** Radius of the gyration (*R*_g_) plot.

The average RMSD values of Sprotein and Sprotein–remdesivir were found to be 1.90 and 2.29 nm, respectively. Remdesivir maintained stable conformation in the active pocket of Sprotein from 25 to 100 ns MD simulations (Figure 8A). It has been found that the structure of Sprotein was largely deviated upon binding of remdesivir. This indicates that binding of remdesivir deviated the structure conformation of Sprotein. The residual and atomic fluctuations were also reported due to binding of remdesivir (Figures 8B,C). The average *R*_*g*_ values for Sprotein and Sprotein–remdesivir were found to be 4.18 and 3.93 nm, respectively (Figure 8D). It was found that Sprotein–remdesivir has more tight packing than Sprotein alone. This might be due to strong binding of remdesivir with Sprotein.

The average SASA values with respect to protein for Sprotein and Sprotein–remdesivir were found to be 513.80 and 473.77 nm^2^, respectively. The SASA plot suggested that internal residues in Sprotein are not exposed to solvent when remdesivir binds to it. Furthermore, the free energy of solvation for Sprotein and Sprotein–remdesivir was found to be 829.97 and 656.94 kJ/mol/nm^2^, respectively. It was found that the total average residues that contributed to structure development in case of Sprotein and Sprotein–remdesivir were found to be 59 and 61%, respectively. It has been found that Sprotein is not unfolded upon binding of remdesivir. It was also found that the volume of Sprotein and Sprotein–remdesivir was 186.77 and 174.05 nm^3^, respectively. The average density of Sprotein and Sprotein–remdesivir was found to be 944.20 and 1,013.23 g/L, respectively (Supplementary Figure S7).

### Hydrogen Bonding

**FIGURE 9:**
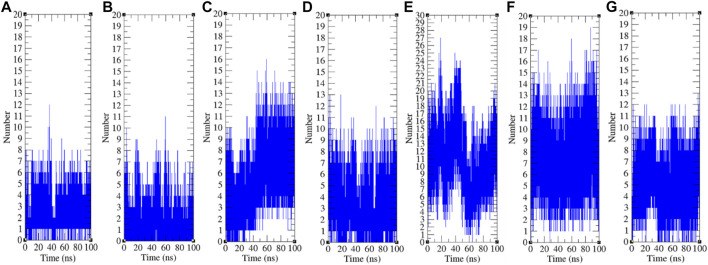
Hydrogen bond analysis. Hydrogen bonds between remdesivir and **(A)** CTD, **(B)** Eprotein, **(C)** Mprotease, **(D)** Mprotein, **(E)** NTD, **(F)** RDRP, and **(G)** Sprotein, respectively.

The hydrogen bond is a significant factor in stabilizing protein conformations. To check the hydrogen bond formations between remdesivir and SARS-CoV-2 proteins such as CTD, Eprotein, Mprotease, Mprotein, NTD, RDRP, and Sprotein, the hydrogen bonds paired within 0.35 nm were estimated during the 100 ns MD simulations. The average number of hydrogen bonds between remdesivir and CTD, Eprotein, Mprotease, Mprotein, NTD, RDRP, and Sprotein was found to be 3, 2, 5, 4, 11, 8, and 5, respectively (Figure 9). The maximum number of hydrogen bonds was found between remdesivir and NTD. RDRP, Sprotein, and Mprotease also showed extensive hydrogen bonding with remdesivir. Weak interactions were reported in case of CTD, Eprotein, and Mprotein.

### Principal Component Analysis

**FIGURE 10:**
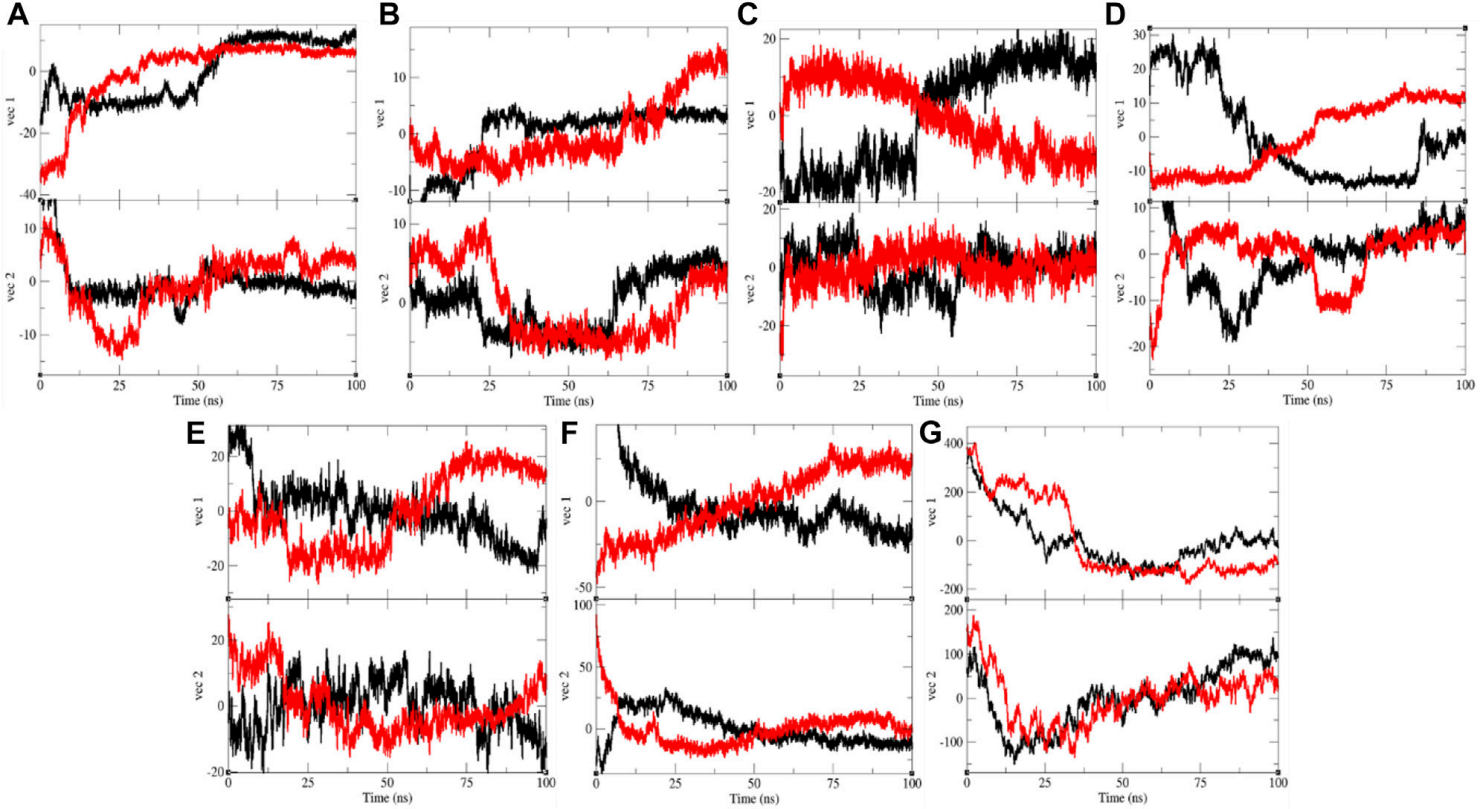
Projection of eigenvectors and components. Projections of trajectories on eigenvectors for **(A)** CTD (black) and CTD–remdesivir (red), **(B)** Eprotein (black) and Eprotein–remdesivir (red), **(C)** Mprotease (black) and Mprotease–remdesivir (red), **(D)** Mprotein (black) and Mprotein–remdesivir (red), **(E)** NTD (black) and NTD–remdesivir (red), **(F)** RDRP (black) and RDRP–remdesivir (red), and **(G)** Sprotein (black) and Sprotein–remdesivir (red), respectively.

The PCA or ED shows an overall expansion of CTD, Eprotein, Mprotease, Mprotein, NTD, RDRP, and Sprotein during MD simulation. It categorizes average atomic motions of CTD, Eprotein, Mprotease, Mprotein, NTD, RDRP, and Sprotein. The entirety of the eigenvalues is a degree of the global mobility in the system to relate the elasticity of a protein. The trace of the covariance matrix and eigenvalues were found to be 192.95, 257.40, 84.53, 95.33, 383.19, 233.40, 450.76, 250.48, 331.98, 440.60, 1,387.72, 1,235.44, 21,461.90, and 33,703.80 nm^2^, for CTD, CTD–remdesivir, Eprotein, Eprotein–remdesivir, Mprotease, Mprotease–remdesivir, Mprotein, Mprotein–remdesivir, NTD, NTD–remdesivir, RDRP, RDRP–remdesivir, Sprotein, and Sprotein–remdesivir, respectively. The trace of the covariance matrix and eigenvalues were found to be more in case of CTD–remdesivir, Eprotein–remdesivir, NTD–remdesivir, and Sprotein–remdesivir than CTD, Eprotein, NTD, and Sprotein, respectively. The higher eigenvalues and the trace of the covariance matrix of CTD–remdesivir, Eprotein–remdesivir, NTD–remdesivir, and Sprotein–remdesivir advise that the average casual fluctuations are more upon binding of remdesivir with CTD, Eprotein, NTD, and Sprotein, respectively. The trace of the covariance matrix and eigenvalues were found to be low in case of Mprotease–remdesivir, Mprotein–remdesivir, and RDRP–remdesivir than Mprotease, Mprotein, and RDRP, respectively. Binding of remdesivir to Mprotease, Mprotein, and RDRP reduces the average motion in protein. This might be due to strong binding of remdesivir. The comprehensive multi-dimensional covariance matrix for individual atom pair covariance and the predictions of trajectories on eigenvectors are described in Figure 10.

### Gibbs Free Energy Landscape

**FIGURE 11:**
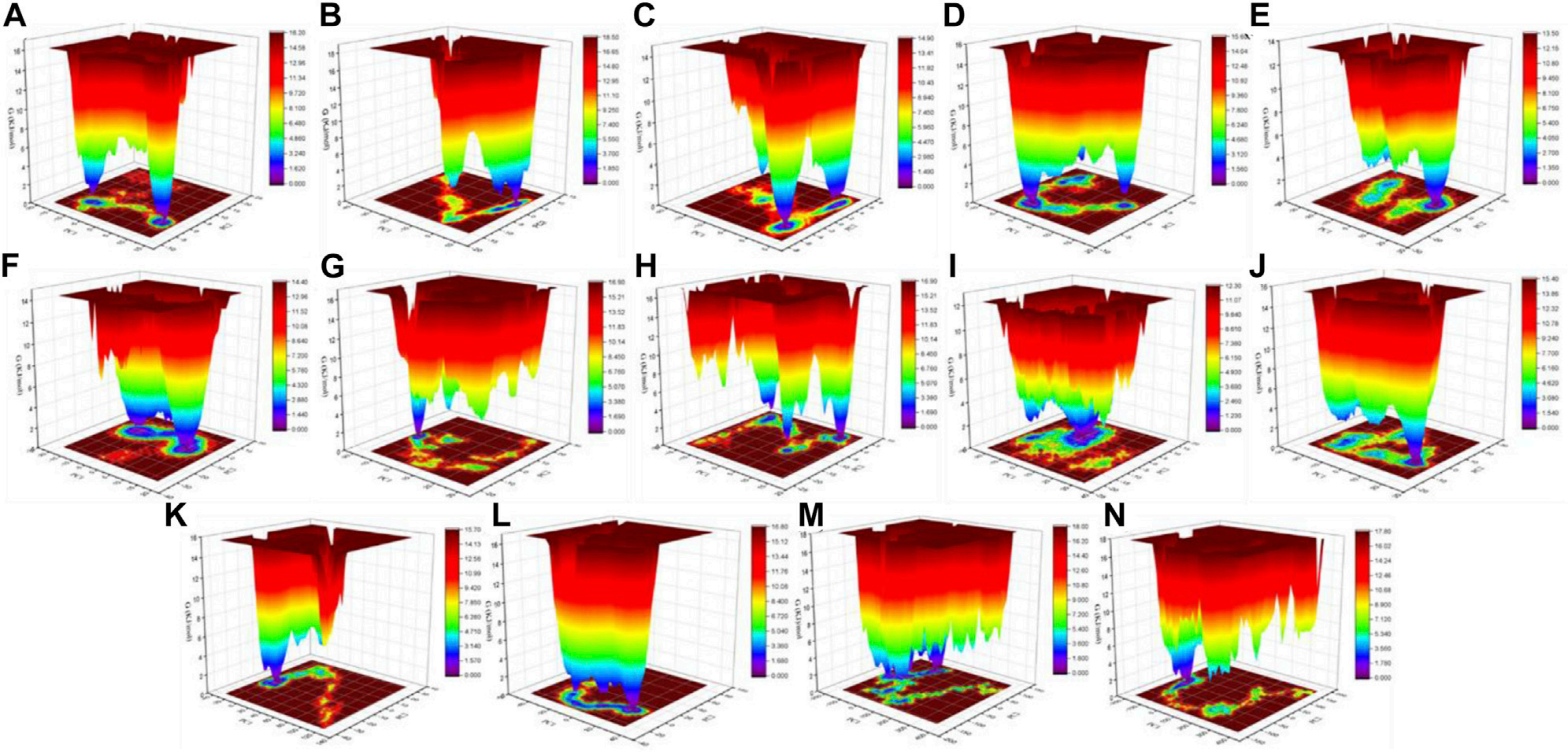
Gibbs energy landscape. Gibbs free energy landscape plot obtained during 100 ns MD simulations for **(A)** CTD, **(B)** CTD–remdesivir, **(C)** Eprotein, **(D)** Eprotein–remdesivir, **(E)** Mprotease, **(F)** Mprotease–remdesivir, **(G)** Mprotein, **(H)** Mprotein–remdesivir, **(I)** NTD, **(J)** NTD–remdesivir, **(K)** RDRP, **(L)** RDRP–remdesivir, **(M)** Sprotein, and **(N)** Sprotein–remdesivir, respectively.

The color-coded energy landscape displayed varied forms for CTD, CTD–remdesivir, Eprotein, Eprotein–remdesivir, Mprotease, Mprotease–remdesivir, Mprotein, Mprotein–remdesivir, NTD, NTD–remdesivir, RDRP, RDRP–remdesivir, Sprotein, and Sprotein–remdesivir, respectively (Figure 11). Each atom pair covariance shows dissimilar outlines in each case. The conforming energy contour records with profound blue gloom signify the lower energy position. Additional blue zones designate transitions in the protein conformation trailed by thermodynamically more favorable regions. The free energy state in the global free energy minimum region of CTD, CTD–remdesivir, Eprotein, Eprotein–remdesivir, Mprotease, Mprotease–remdesivir, Mprotein, Mprotein–remdesivir, NTD, NTD–remdesivir, RDRP, RDRP–remdesivir, Sprotein, and Sprotein–remdesivir is different in each case. A comparison between the full views of the Gibbs free energy values of CTD, CTD–remdesivir, Eprotein, Eprotein–remdesivir, Mprotease, Mprotease–remdesivir, Mprotein, Mprotein–remdesivir, NTD, NTD–remdesivir, RDRP, RDRP–remdesivir, Sprotein, and Sprotein–remdesivir suggested that these systems have different outlines of global minima. There are more changes in the global minima patterns in case of Mprotease, Mprotein, and RDRP when remdesivir binds to them. In case of Mprotease and RDRP, the global minima changed from single minima to dark blue color when remdesivir bound to them. In case of Mprotein, there was single deep blue well, and it shifted to three global minima in case of Mprotein–remdesivir.

### MMPBSA Analysis

The free energy for binding the ligand to the protein receptor was mined by executing the MMPBSA scheme (Kumari et al., 2014). It is calculated by using polar and apolar solvation constraints to obtain the energies linked with binding of remdesivir to CTD, Eprotein, Mprotease, Mprotein, NTD, RDRP, and Sprotein during MD simulations. The point of these calculations is to attain van der Waals and electrostatic interactions and net non-bonded potential energy between remdesivir and CTD, Eprotein, Mprotease, Mprotein, NTD, RDRP, and Sprotein. The MMPBSA calculations also suggested that remdesivir has strong binding affinity with Mprotein, Mprotease, and RDRP. It has been found that the average binding free energy between remdesivir and Mprotein, Mprotease, and RDRP was −454.69 ± 29.11 kJ/mol, −357.78 ± 39.53 kJ/mol, and −245.12 ± 45.12 kJ/mol, respectively. The MMPBSA calculations also suggested that remdesivir shows strong interactions with these proteins. The previous *in vitro* experiment showed that the EC_50_ value of remdesivir for SARS-CoV-2 is 0.77 μM (Wang M. et al., 2020). Nguyen et al. estimated the binding free energy for Mprotease–remdesivir and RDRP–remdesivir using umbrella sampling. They found that the binding free energy for Mprotease–remdesivir and RDRP–remdesivir was −8.69 ± 0.36 kcal/mol (−36.36 kJ/mol) and −9.34 ± 0.38 kcal/mol (−39.08 kJ/mol), respectively (Nguyen et al., 2020). The huge difference between binding free energies is due to different methods used in both the cases. Although the computational results have several limitations, it can be used for comparative analysis.

## Conclusion

By means of several computational tools, the association of remdesivir with the C-terminal domain of SARS-CoV-2 nucleocapsid protein, envelope protein, main protease, membrane protein, N-terminal domain of nucleocapsid phosphoprotein, spike protein, and RNA-dependent RNA polymerase of SARS-CoV-2 has been studied. It has been found that together with RNA-dependent RNA polymerase, the membrane protein and main protease of SARS-CoV-2 are also targets for remdesivir. In this study, we analyzed the possible inhibitory mechanism of remdesivir against SARS-CoV-2 using electrostatic interactions, structural deviations, solvent-accessible surface area, secondary structure, principal component analysis, and MMPBSA approaches. Although we have performed detailed computational analysis of binding remdesivir to CTD, Eprotein, Mprotease, Mprotein, NTD, RDRP, and Sprotein, there are many possible research limitations. The experimental validations of the present work are needed to support these data. The information on the different targets might be beneficial for the development of potential drugs.

## Data Availability

The raw data supporting the conclusion of this article will be made available by the authors, without undue reservation, to any qualified researcher.
