# Erratum: Remdesivir strongly binds to RNA-dependent RNA polymerase, membrane protein, and main protease of SARS-CoV-2: Indication from molecular modeling and simulations

**DOI:** 10.3389/fphar.2022.1027099

**Published:** 2022-09-16

**Authors:** Frontiers Production Office

**Affiliations:** Frontiers Media SA, Lausanne, Switzerland

**Keywords:** SARS-CoV-2, remdesivir, main protease, membrane proteins, RNA-dependent RNA polymerase

Due to a production error, the figures were incorrectly matched with their captions in the final published article. The correct figures and captions appear below.

The publisher apologizes for this mistake. The original version of this article has been updated.

**FIGURE 1:**
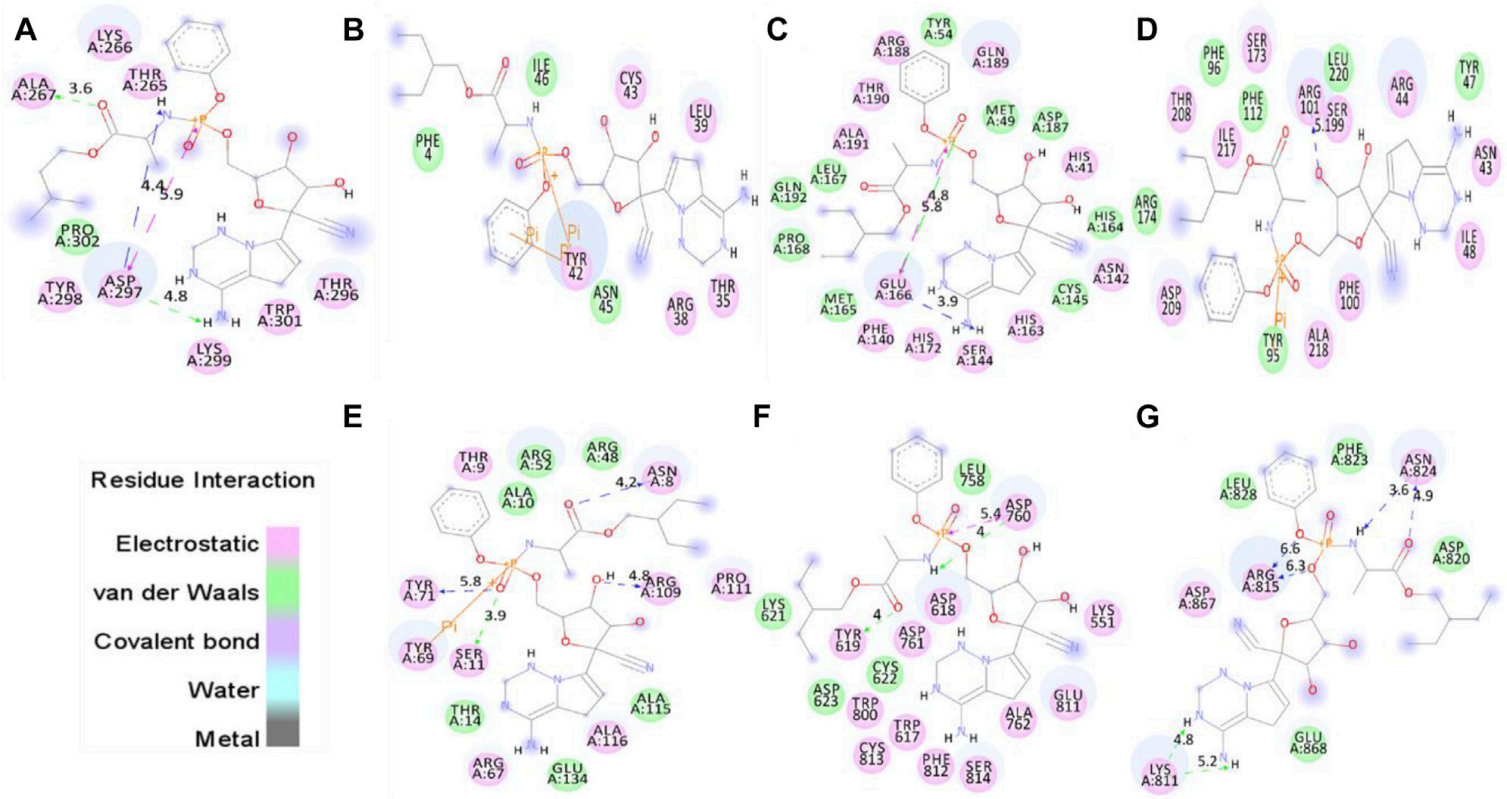
The molecular docking of remdesivir into the active pocket of CTD, Eprotein, Mprotease, Mprotein, NTD, RDP, and Sprotein, respectively. The structure indicated different residual interactions in **(A)** CTD-remdesivir, **(B)** Eprotein-remdesivir, **(C)** Mprotease-remdesivir, **(D)** Mprotein-remdesivir, **(E)** NTD-remdesivir, **(F)** RDP-remdesivir, and **(G)** Sprotein-remdesivir, respectively. The electrostatic, van der Waals, and covalent bonds are represented by pink, green, and purple color, respectively.

**FIGURE 2:**
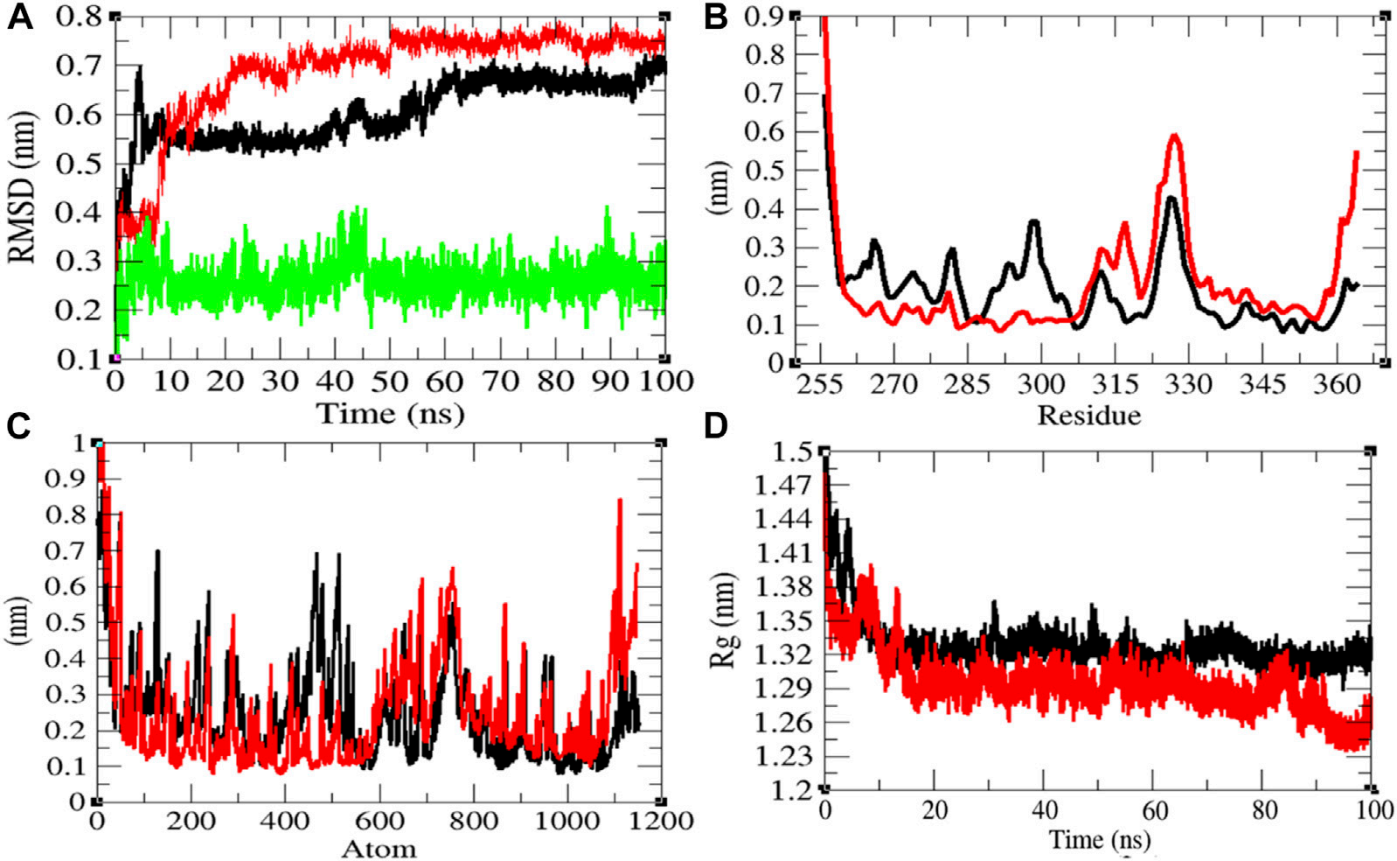
Structural dynamics of CTD. **(A)** Root mean square deviations plot for CTD (black), CTD-remdesivir (red), and remdesivir (green) as a function of time. **(B)** Root mean square fluctuations vs. residues. **(C)** Root mean square fluctuations vs. atoms. **(D)** Radius of gyration (*R*_g_) plot.

**FIGURE 3:**
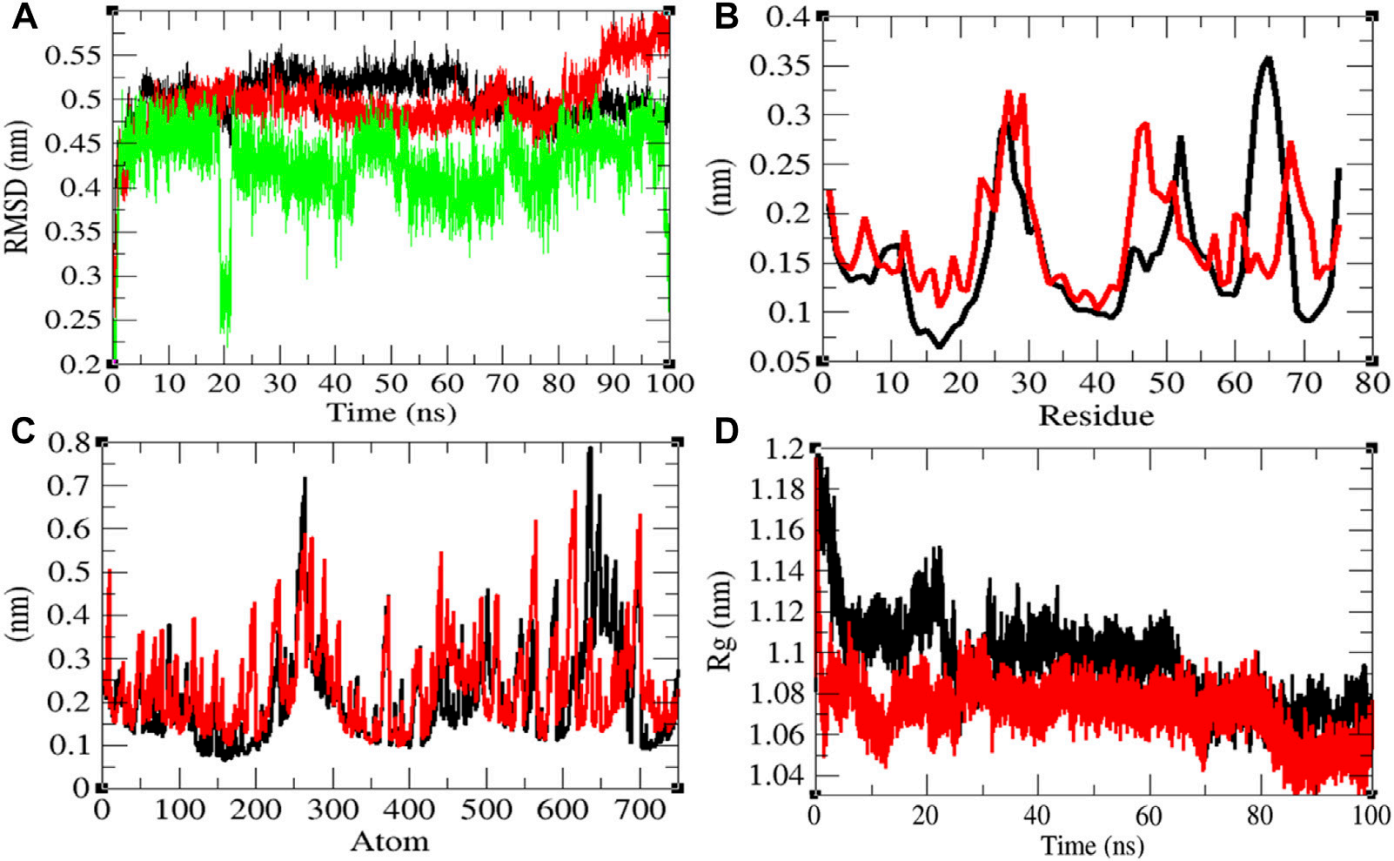
Structural dynamics of Eprotein. **(A)** Root mean square deviations plot for Eprotein (black), Eprotein-remdesivir (red), and remdesivir (green) as a function of time. **(B)** Root mean square fluctuations vs. residues. **(C)** Root mean square fluctuations vs. atoms. **(D)** Radius of gyration (*R*_g_) plot.

**FIGURE 4:**
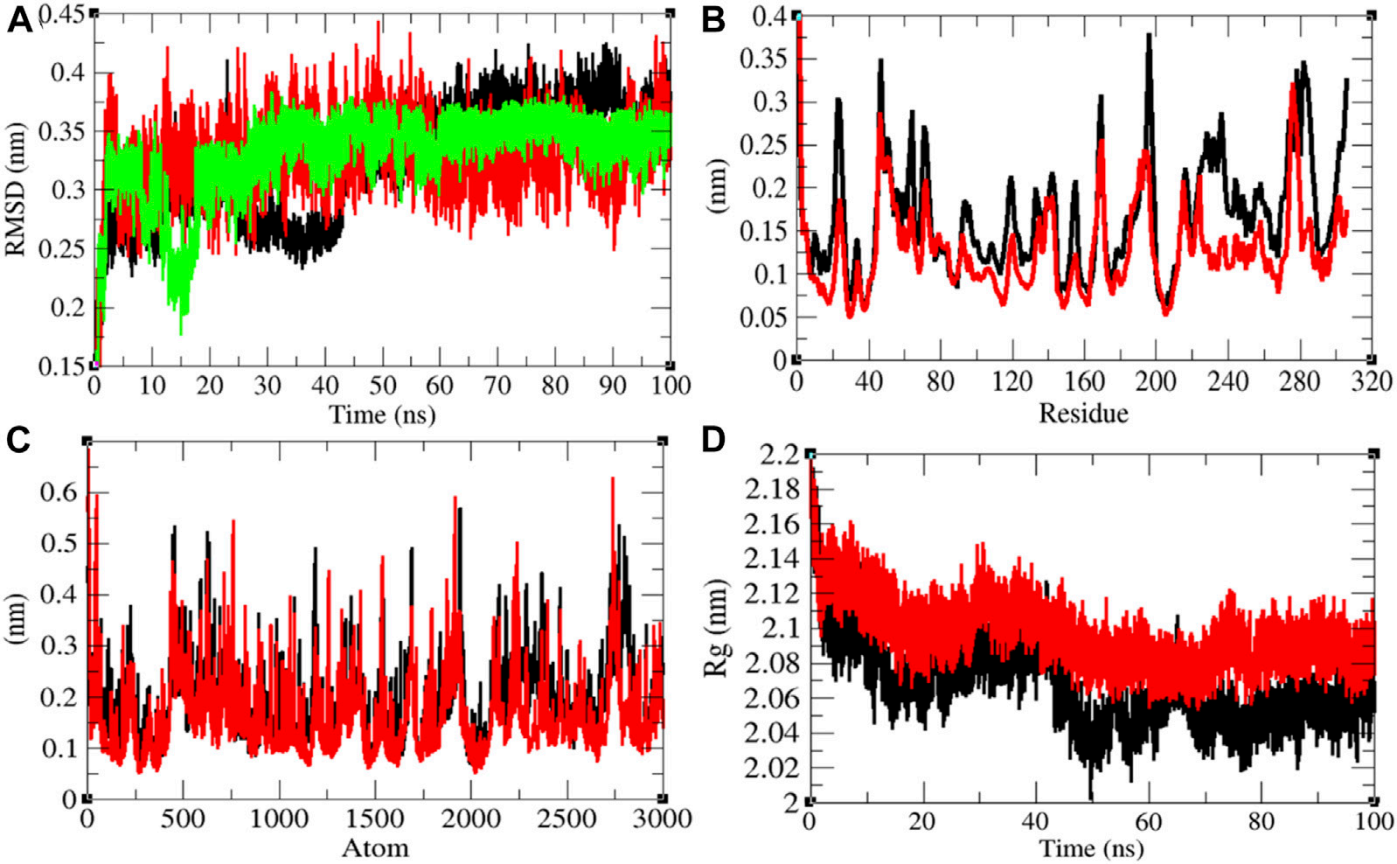
Structural dynamics of Mprotease. **(A)** Root mean square deviations plot for Mprotease (black), Mprotease-remdesivir (red), and remdesivir (green) as a function of time. **(B)** Root mean square fluctuations vs. residues. **(C)** Root mean square fluctuations vs. atoms. **(D)** Radius of gyration (*R*_g_) plot.

**FIGURE 5:**
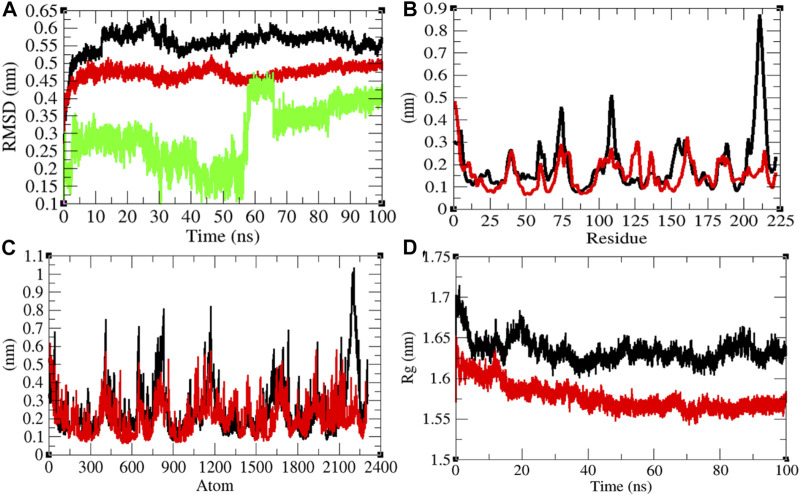
Structural dynamics of Mprotein. **(A)** Root mean square deviations plot for Mprotein (black), Mprotein-remdesivir (red), and remdesivir (green) as a function of time. **(B)** Root mean square fluctuations vs. residues. **(C)** Root mean square fluctuations vs. atoms. **(D)** Radius of gyration (*R*_g_) plot.

**FIGURE 6:**
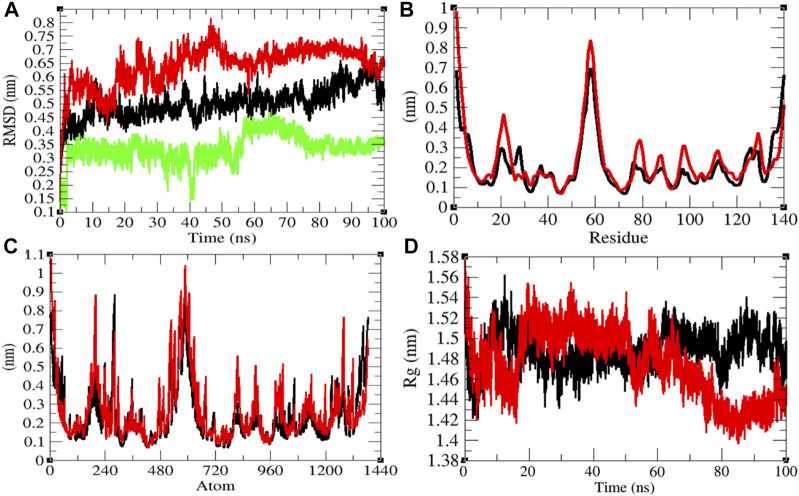
Structural dynamics of NTD. **(A)** Root mean square deviations plot for NTD (black), NTD -remdesivir (red), and remdesivir (green) as a function of time. **(B)** Root mean square fluctuations vs. residues. **(C)** Root mean square fluctuations vs. atoms. **(D)** Radius of gyration (*R*_g_) plot.

**FIGURE 7:**
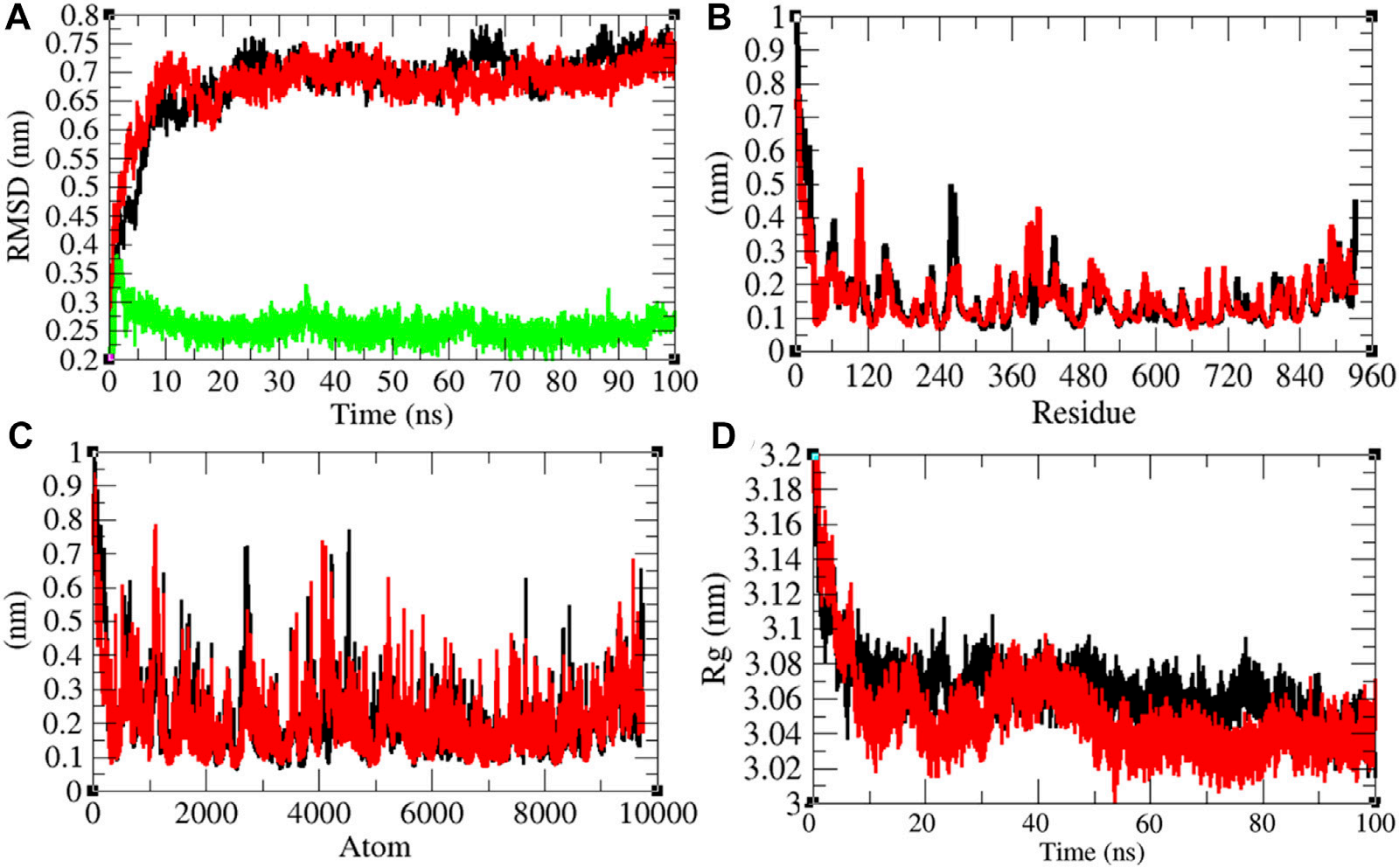
Structural dynamics of RDRP. **(A)** Root mean square deviations plot for RDRP (black), RDRP-remdesivir (red), and remdesivir (green) as a function of time. **(B)** Root mean square fluctuations vs. residues. **(C)** Root mean square fluctuations vs. atoms. **(D)** Radius of gyration (*R*_g_) plot.

**FIGURE 8:**
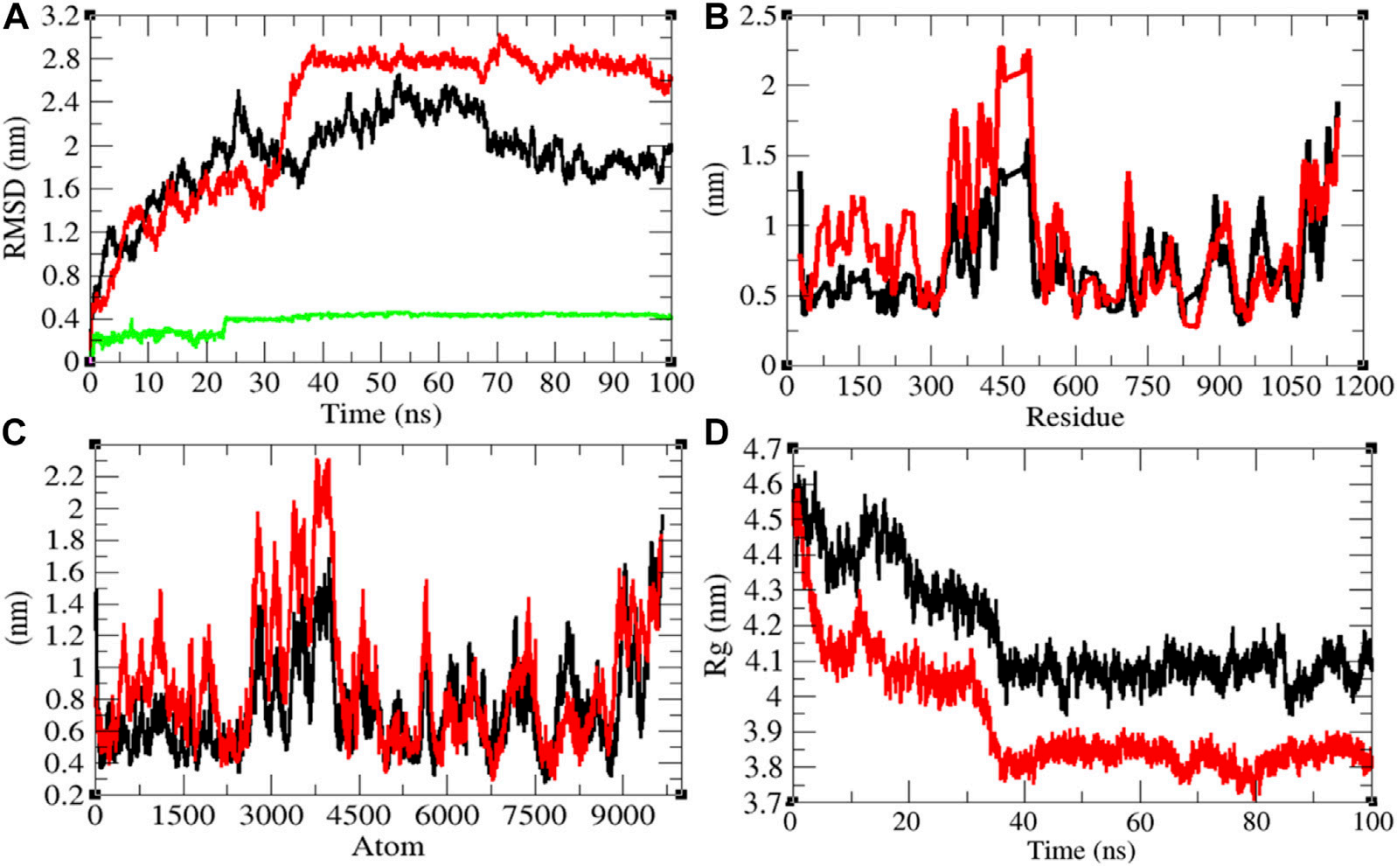
Structural dynamics of Sprotein. **(A)** Root mean square deviations plot for Sprotein (black), Sprotein-remdesivir (red), and remdesivir (green) as a function of time. **(B)** Root mean square fluctuations vs. residues. **(C)** Root mean square fluctuations vs. atoms. **(D)** Radius of gyration (*R*_g_) plot.

**FIGURE 9:**
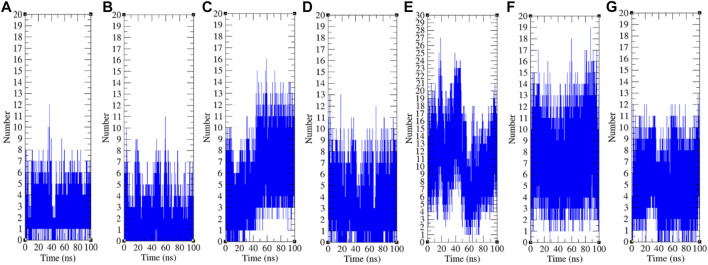
Hydrogen bonds analysis. The hydrogen bonds between remdesivir and **(A)** CTD, **(B)** Eprotein, **(C)** Mprotease, **(D)** Mprotein, **(E)** NTD, **(F)** RDRP, and **(G)** Sprotein, respectively.

**FIGURE 10:**
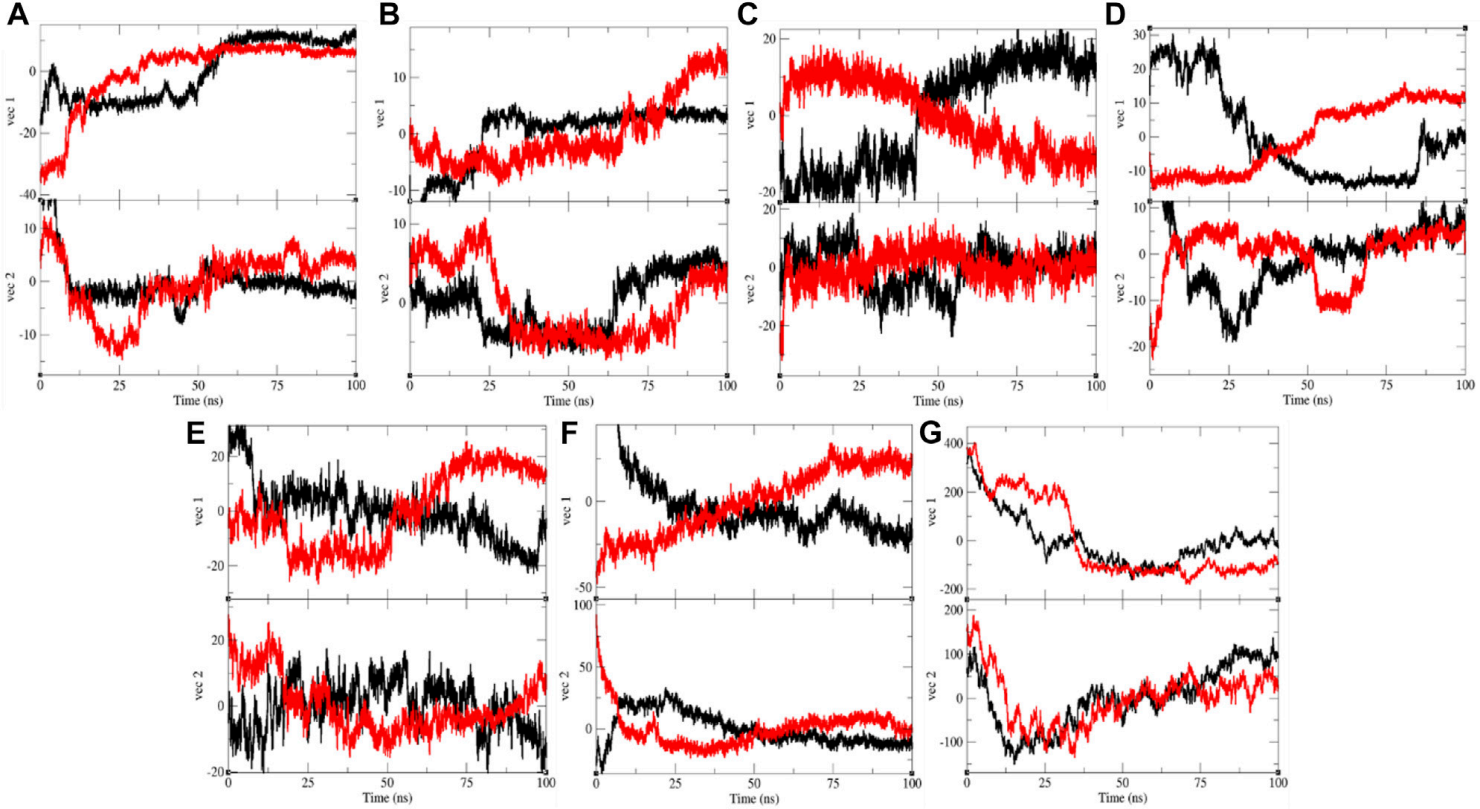
Projection of eigenvectors and components. The projections of trajectories on eigenvectors for **(A)** CTD (black) and CTD-remdesivir (red), **(B)** Eprotein (black) and Eprotein-remdesivir (red), **(C)** Mprotease (black) and Mprotease-remdesivir (red), **(D)** Mprotein (black) and Mprotein-remdesivir (red), **(E)** NTD (black) and NTD-remdesivir (red), **(F)** RDRP (black) and RDRP-remdesivir (red), and **(G)** Sprotein (black), and Sprotein-remdesivir (red), respectively.

**FIGURE 11:**
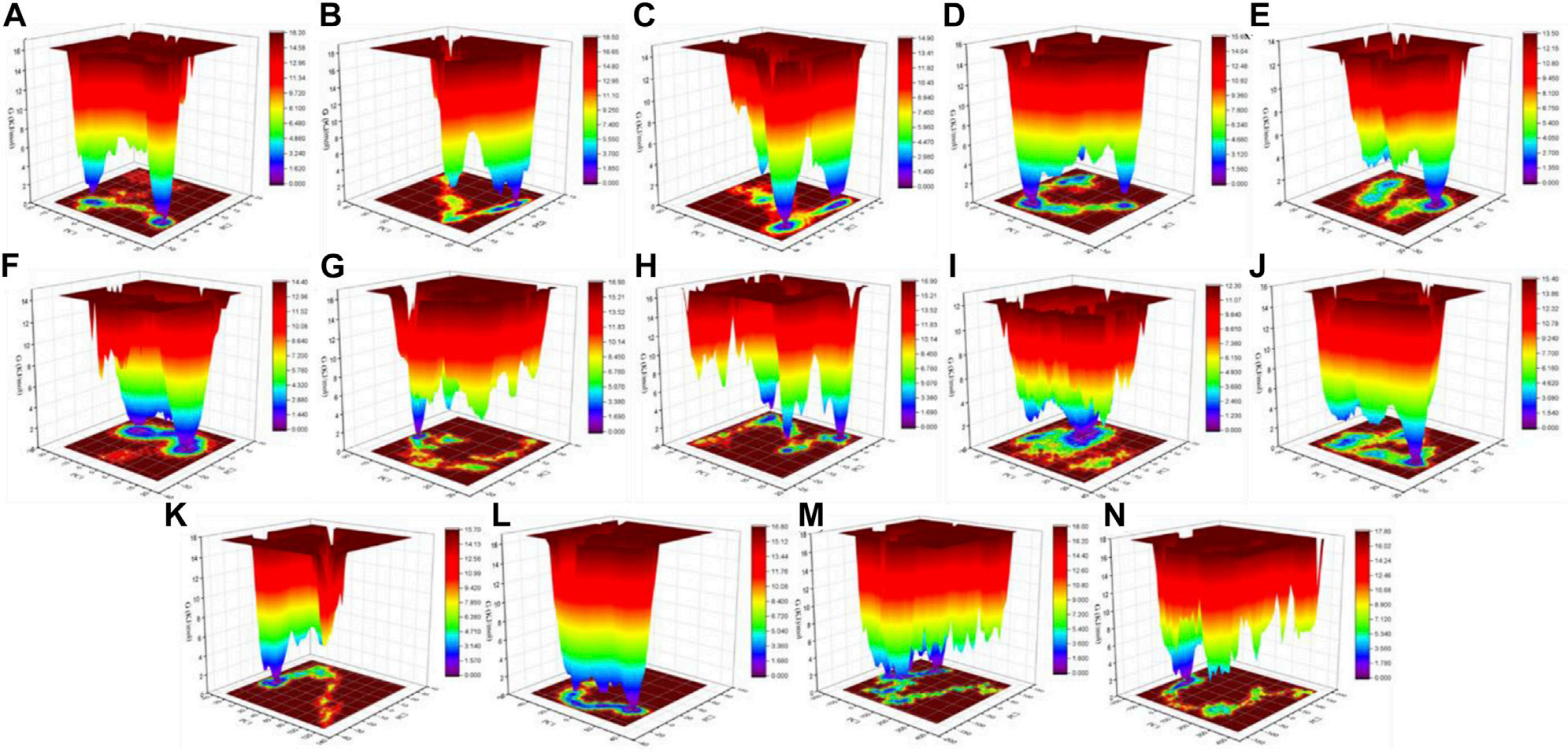
Gibbs energy landscape. The Gibbs energy landscape plot obtained during 100 ns MD simulations for **(A)** CTD, **(B)** CTD-remdesivir, **(C)** Eprotein, **(D)** Eprotein-remdesivir, **(E)** Mprotease, **(F)** Mprotease-remdesivir, **(G)** Mprotein, **(H)** Mprotein-remdesivir, **(I)** NTD, **(J)** NTD-remdesivir, **(K)** RDRP, **(L)** RDRP-remdesivir, **(M)** Sprotein, and **(N)** Sprotein-remdesivir, respectively.

