# Supplementary material for: Remdesivir Strongly Binds to RNA-Dependent RNA Polymerase, Membrane Protein, and Main Protease of SARS-CoV-2: Indication From Molecular Modeling and Simulations
Source: Front Pharmacol. 2021 Jul 7;12:710778. doi: 10.3389/fphar.2021.710778 (PMC8293383; doi:10.3389/fphar.2021.710778)
Supplement: Supplementary file 1 [file DataSheet1.pdf]

Remdesivir Strongly Binds to RNA-Dependent RNA Polymerase, Membrane protein, and Main Protease of SARS-CoV-2: Indication from Molecular Modelling and Simulations

Faez Iqbal Khan[1#], Tongzhou Kang[1#], Haider Ali[2], and Dakun Lai[1, *]

[1]School of Electronic Science and Engineering, University of Electronic Science and Technology of China, Chengdu, Sichuan, China.
[2]Faculty of medicine, International Ala-too University, Bishkek 720048, Kyrgyzstan.

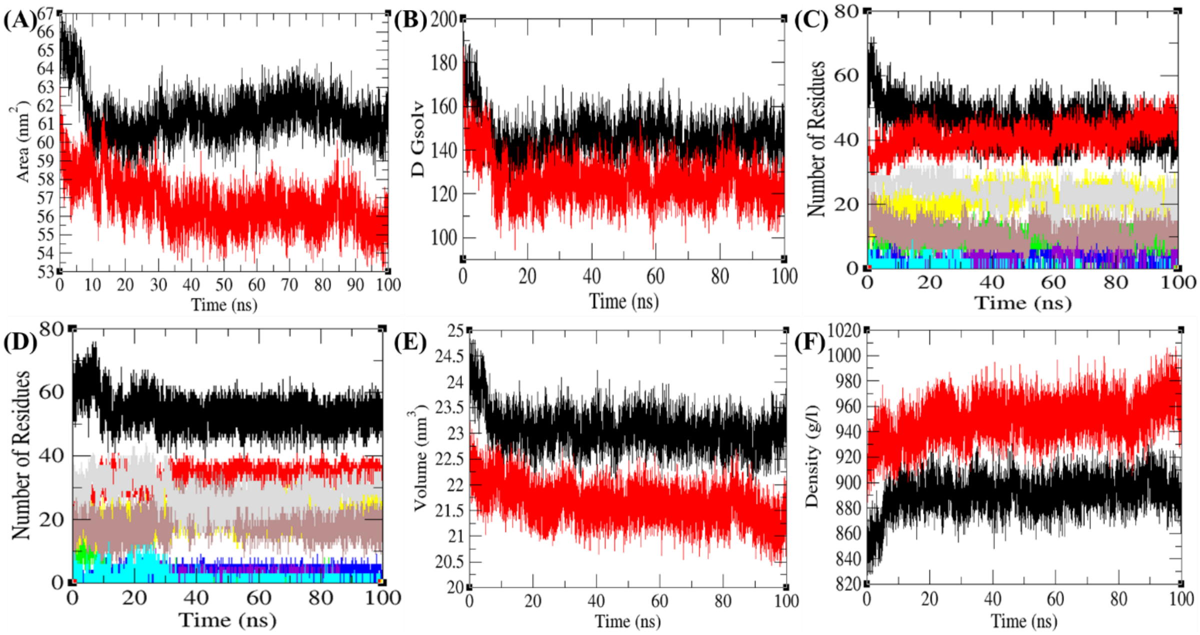

Figure S1. The secondary structure plot of CTD. The SASA plot of **(A)** CTD (black) and **(B)** CTD-remdesivir (red). The graphical representation indicating structural elements present in **(C)** CTD, and **(D)** CTD-remdesivir. The secondary structure elements represent α-helices, β-sheets, β-bridge, and turns. Each structural element has been represented by different colour such as black (overall structure), red (coil), green (β-sheet), blue (β-bridge), yellow (bend), brown (turn), grey (α-helix), and cyan (3-helix), respectively. **(E)** The structural volume, and **(F)** the density of CTD (black) and CTD-remdesivir (red) calculated during MD simulations.

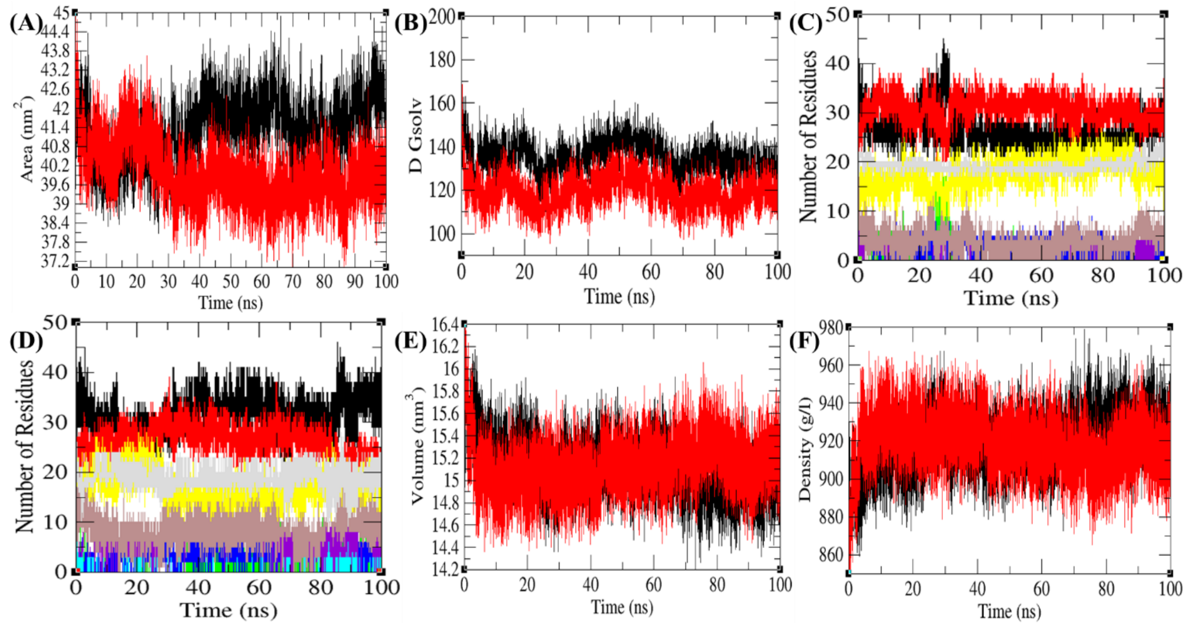

Figure S2. The secondary structure plot of Eprotein. The SASA plot of **(A)** Eprotein (black) and **(B)** Eprotein -remdesivir (red). The graphical representation indicating structural elements present in **(C)** Eprotein, and **(D)** Eprotein -remdesivir. **(E)** The structural volume and **(F)** the density of Eprotein (black) and Eprotein -remdesivir (red) calculated during MD simulations. The colour of the secondary structure plot has the same meaning as in the previous figure.

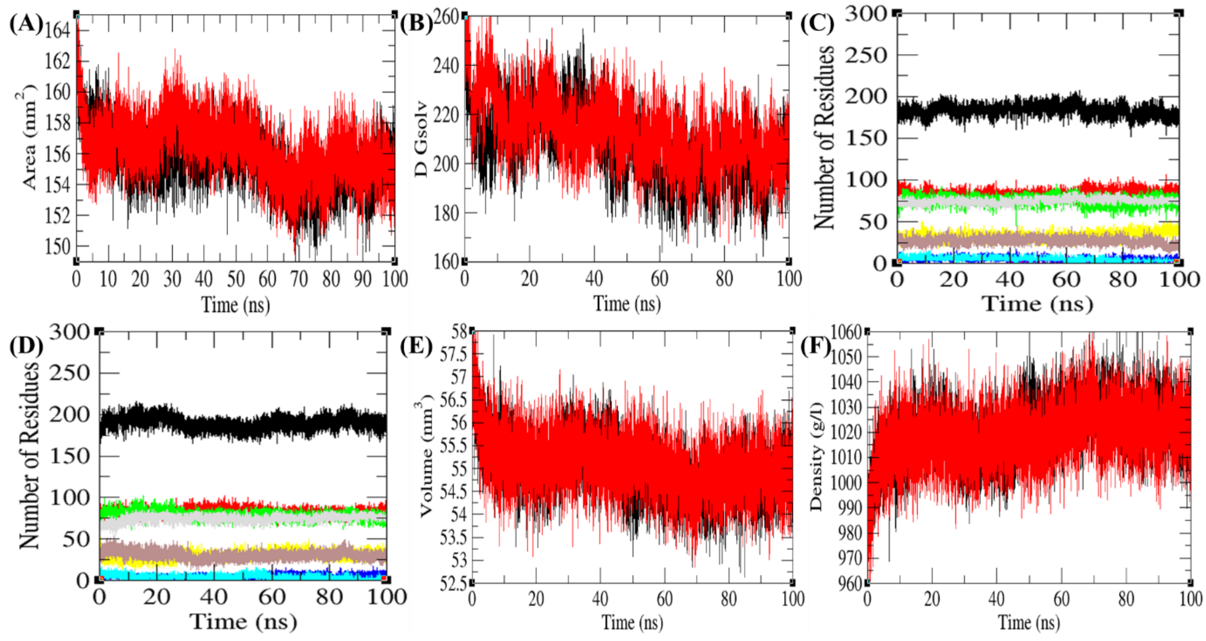

Figure S3. The secondary structure plot of Mprotease. The SASA plot of **(A)** Mprotease (black) and **(B)** Mprotease -remdesivir (red). The graphical representation indicating structural elements present in **(C)** Mprotease, and **(D)** Mprotease -remdesivir. **(E)** The structural volume and **(F)** the density of Mprotease (black) and Mprotease -remdesivir (red) calculated during MD simulations. The colour of the secondary structure plot has the same meaning as in the previous figure.

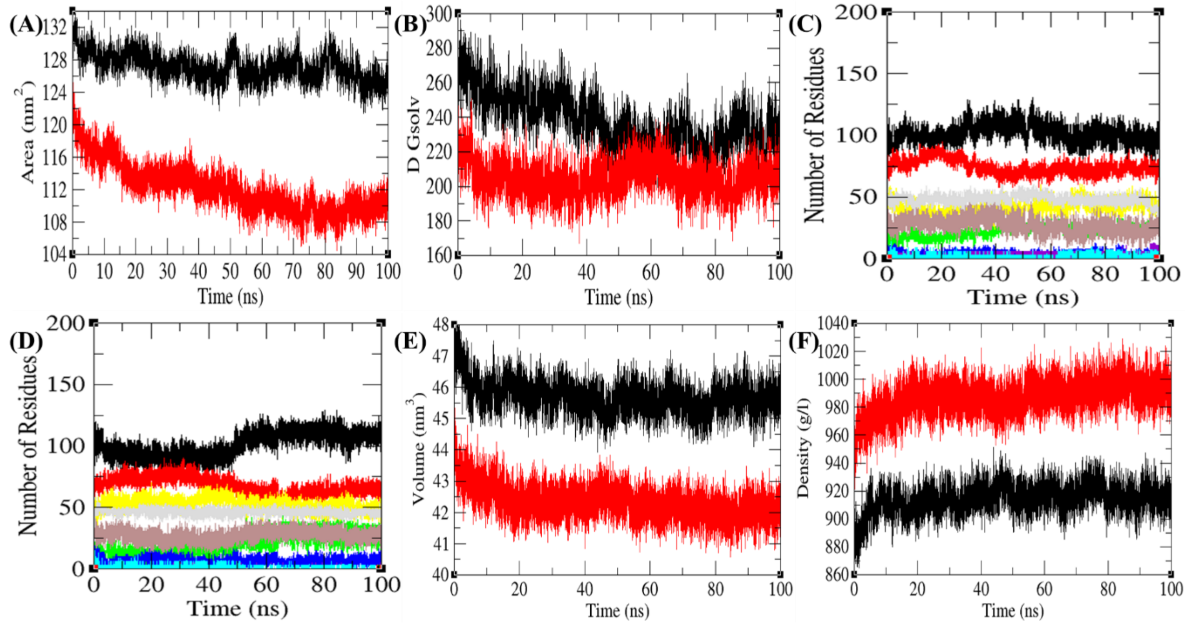

Figure S4. The secondary structure plot of Mprotein. The SASA plot of **(A)** Mprotein (black) and **(B)** Mprotein -remdesivir (red). The graphical representation indicating structural elements present in **(C)** Mprotein, and **(D)** Mprotein -remdesivir. **(E)** The structural volume and **(F)** the density of Mprotein (black) and Mprotein-remdesivir (red) calculated during MD simulations. The colour of the secondary structure plot has the same meaning as in the previous figure.

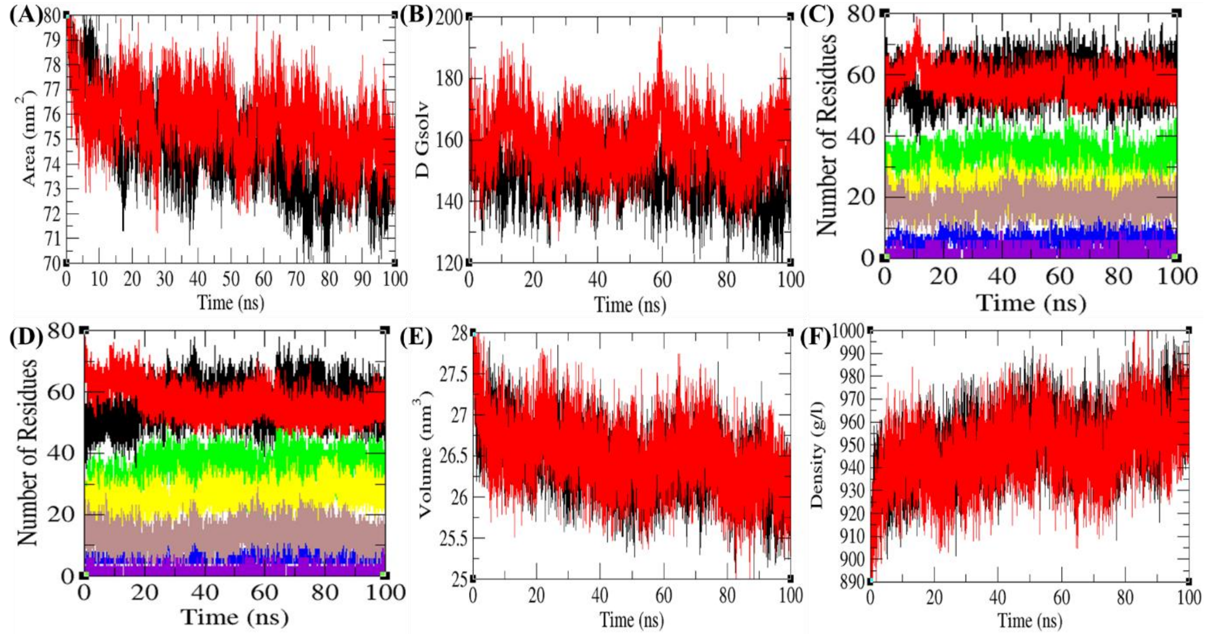

Figure S5. The secondary structure plot of NTD. The SASA plot of **(A)** NTD (black) and **(B)** NTD -remdesivir (red). The graphical representation indicating structural elements present in **(C)** NTD, and **(D)** NTD -remdesivir. **(E)** The structural volume and **(F)** the density of NTD (black) and NTD -remdesivir (red) calculated during MD simulations. The colour of the secondary structure plot has the same meaning as in the previous figure.

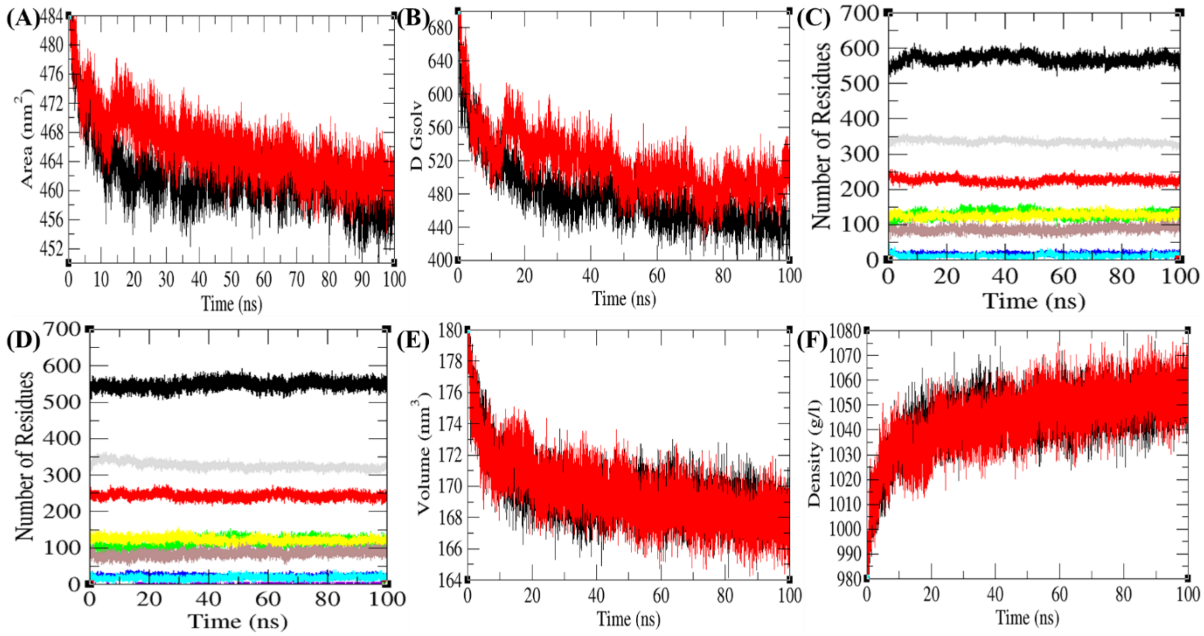

Figure S6. The secondary structure plot of RDRP. The SASA plot of **(A)** RDRP (black) and **(B)** RDRP -remdesivir (red). The graphical representation indicating structural elements present in **(C)** RDRP, and **(D)** RDRP -remdesivir. **(E)** The structural volume and **(F)** the density of RDRP (black) and RDRP-remdesivir (red) calculated during MD simulations. The colour of the secondary structure plot has the same meaning as in the previous figure.

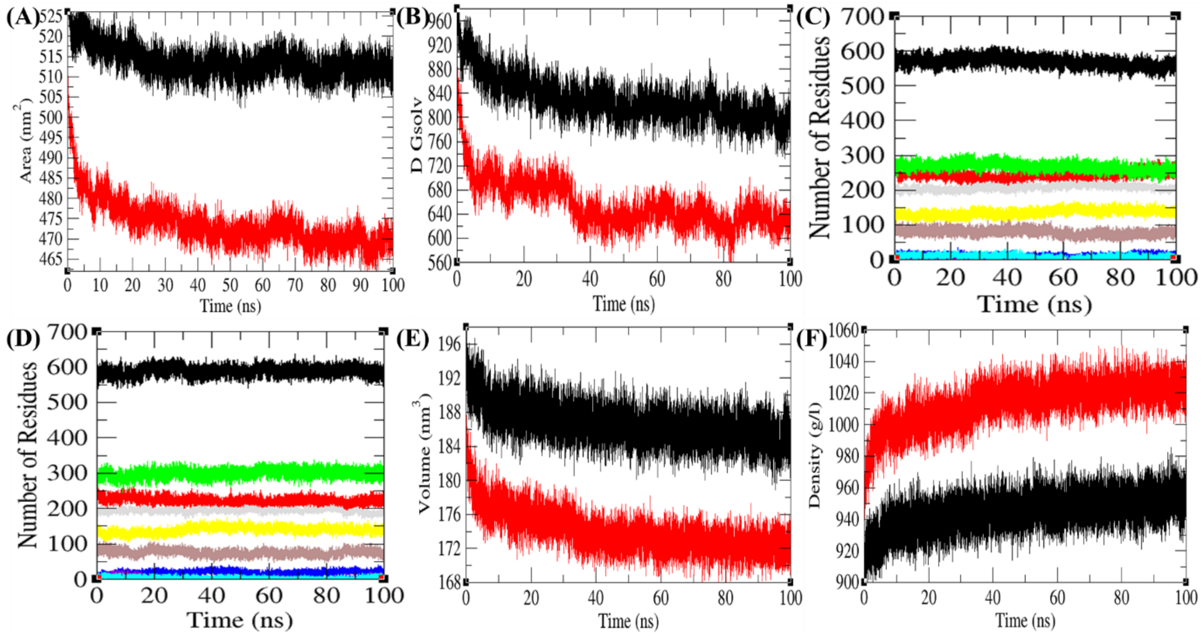

Figure S7. The secondary structure plot of Sprotein. The SASA plot of **(A)** Sprotein (black) and **(B)** Sprotein -remdesivir (red). The graphical representation indicating structural elements present in **(C)** Sprotein, and **(D)** Sprotein -remdesivir. **(E)** The structural volume and **(F)** the density of Sprotein (black) and Sprotein -remdesivir (red) calculated during MD simulations. The colour of the secondary structure plot has the same meaning as in the previous figure.
